# Deciphering the Biotic and Climatic Factors That Influence Floral Scents: A Systematic Review of Floral Volatile Emissions

**DOI:** 10.3389/fpls.2020.01154

**Published:** 2020-07-31

**Authors:** Gerard Farré-Armengol, Marcos Fernández-Martínez, Iolanda Filella, Robert R. Junker, Josep Peñuelas

**Affiliations:** ^1^Department of Biosciences, University of Salzburg, Salzburg, Austria; ^2^CSIC, Global Ecology Unit CREAF-CSIC-UAB, Barcelona, Spain; ^3^CREAF, Barcelona, Spain; ^4^PLECO (Plants and Ecosystems), Department of Biology, University of Antwerp, Wilrijk, Belgium; ^5^Evolutionary Ecology of Plants, Department of Biology, Philipps-University Marburg, Marburg, Germany

**Keywords:** climate, floral volatiles, phylogeny, pollination syndromes, terpenoids, VOC composition, VOC richness

## Abstract

Currently, a global analysis of the information available on the relative composition of the floral scents of a very diverse variety of plant species is missing. Such analysis may reveal general patterns on the distribution and dominance of the volatile compounds that form these mixtures, and may also allow measuring the effects of factors such as the phylogeny, pollination vectors, and climatic conditions on the floral scents of the species. To fill this gap, we compiled published data on the relative compositions and emission rates of volatile organic compounds (VOCs) in the floral scents of 305 plant species from 66 families. We also gathered information on the groups of pollinators that visited the flowers and the climatic conditions in the areas of distribution of these species. This information allowed us to characterize the occurrence and relative abundances of individual volatiles in floral scents and the effects of biotic and climatic factors on floral scent. The monoterpenes trans-β-ocimene and linalool and the benzenoid benzaldehyde were the most abundant floral VOCs, in both ubiquity and predominance in the floral blends. Floral VOC richness and relative composition were moderately preserved traits across the phylogeny. The reliance on different pollinator groups and the climate also had important effects on floral VOC richness, composition, and emission rates of the species. Our results support the hypothesis that key compounds or compounds originating from specific biosynthetic pathways mediate the attraction of the main pollinators. Our results also indicate a prevalence of monoterpenes in the floral blends of plants that grow in drier conditions, which could link with the fact that monoterpene emissions protect plants against oxidative stresses throughout drought periods and their emissions are enhanced under moderate drought stress. Sesquiterpenes, in turn, were positively correlated with mean annual temperature, supporting that sesquiterpene emissions are dominated mainly by ambient temperature. This study is the first to quantitatively summarise data on floral-scent emissions and provides new insights into the biotic and climatic factors that influence floral scents.

## Introduction

Floral scent is an important trait of flowering plants and plays major roles in the interactions of plants with other organisms, including the attraction of pollinators (Raguso, 2004; Schiestl, 2010; Farré-Armengol et al., 2013; Junker and Parachnowitsch, 2015; Kantsa et al., 2018). Effective pollinators (those that carry pollen from the anthers to the stigmas of conspecific plants) are either specialist floral visitors of a limited spectrum of plant species or generalist floral visitors with a short-term specialization known as flower constancy (Chittka et al., 1999). Both pollinators with specialized innate flower preferences and those temporarily specialized *via* associative learning depend on cues or signals to distinguish amongst plant species (Chittka and Thomson, 2001; Kunze and Gumbert, 2001; Chittka and Raine, 2006; Majetic et al., 2008; Burger et al., 2010; Leonard et al., 2011a; Leonard et al., 2011b). Floral volatiles are key floral traits that mediate flower–visitor interactions by attracting pollinators, structuring flower–visitor communities, and defending against plant and flower antagonists (Junker and Blüthgen, 2010; Junker et al., 2010; Galen et al., 2011; Schiestl et al., 2014; Junker and Parachnowitsch, 2015). In addition to pollinator attraction, floral scents play major roles in the interactions with herbivores, parasitoids, and floral larcenists (Junker and Blüthgen, 2008; Raguso, 2008a; Kessler et al., 2008; Junker and Blüthgen, 2010; Galen et al., 2011; Farré-Armengol et al., 2013; Junker, 2016), and they also have important effects on the growth and composition of floral microbial communities (Heil, 2011; Junker et al., 2011; Huang et al., 2012; Junker and Tholl, 2013; Farré-Armengol et al., 2016a).

Pollinators play a major role in the reproduction of most angiosperms (Ollerton et al., 2011) and exert important selection pressures on plant and floral phenotypes, including floral scents (Wright and Schiestl, 2009; Parachnowitsch et al., 2012; Parachnowitsch et al., 2013; Schiestl and Johnson, 2013). The pollination syndrome hypothesis postulates that the floral traits of unrelated plants pollinated by the same pollinators tend to converge, including advertising signals (Faegri and van der Pijl, 1979; Fenster et al., 2004). Researchers have long discussed pollination syndromes, arguing in favour or against their reliability as effective classifiers of floral phenotypes that can be used to predict the plant’s most efficient pollinators (Herrera, 1996; Ollerton, 1996; Waser et al., 1996; Armbruster et al., 2000; Fenster et al., 2004; Lázaro et al., 2008; Raguso, 2008b; Ollerton et al., 2009; Rosas-Guerrero et al., 2014; Ollerton et al., 2015). Many studies have described cases of floral-trait convergence by mono- and polyphyletic groups of plant species that share their main pollinators (Thomson et al., 2000; Stuurman et al., 2004; Wilson et al., 2004; Rosas-Guerrero et al., 2014). Some studies have reported convergent evolution in floral-scent composition driven by a shared reliance on the same pollinator group (Knudsen and Tollsten, 1993; Knudsen and Tollsten, 1995; Miyake et al., 1998; Andersson et al., 2002; Knudsen et al., 2004). Notable evidence also suggests that pollinators have strong evolutionary impacts on the intensity and composition of floral scents emitted by plants (Parachnowitsch et al., 2012; Parachnowitsch et al., 2013).

Plant emissions of volatile organic compounds (VOCs), including floral scents, can be affected by climatic variables such as temperature and humidity and by other environmental abiotic variables such as light, CO_2_ concentration, wind speed, or the concentration of diverse oxidative pollutants such as ozone and nitrogen oxides. The effects of all these environmental abiotic variables and stresses on foliar VOC emissions and on VOC emissions from vegetation at a global scale are well characterized (Kesselmeier and Staudt, 1999; Peñuelas and Llusià, 2001; Owen et al., 2002; Niinemets et al., 2004; Duhl et al., 2008; Niinemets et al., 2010; Holopainen and Gershenzon, 2010; Niinemets, 2010; Peñuelas and Staudt, 2010), as well as those of endogenous variables that are partially controlled by the environment, such as plant nutrient contents (Fernández-Martínez et al., 2018 and references therein). Few studies, though, have addressed the effects of climatic variables on floral-scent emissions. Some of these studies have shed some light on the responses of floral volatile emissions to temperature (Jacobsen and Olsen, 1994; Sagae et al., 2008; Hu et al., 2013; Farré-Armengol et al., 2014; Farré-Armengol et al., 2015a), drought (Burkle and Runyon, 2016; Glenny et al., 2018), light (Jacobsen and Olsen, 1994; Hu et al., 2013), and pollution (Girling et al., 2013; Lusebrink et al., 2015; Farré-Armengol et al., 2016b; Saunier and Blande, 2019). Environmental variables have such effects on floral-scent emissions, so we hypothesize that climate can potentially select floral scents with properties that are most suited to the environmental conditions that plants and their flowers experience.

A global analysis of the currently available information on the floral scents of various species from many families is needed to shed light on how factors such as phylogeny, pollinators, and climate determine floral VOC emissions of the species. Previous studies by Knudsen et al. (1993) and Knudsen et al. (2006) qualitatively described the occurrence of >1,700 compounds in the flowers of 991 species and discussed whether the occurrence and richness of particular volatiles had phylogenetic signals and whether the compounds depended on the pollination biology of the species, i.e. their main pollinator type. The available data on the quantitative compositions and emission rates of floral VOCs, however, have not yet been compiled and analyzed. We aimed to fill this gap by searching published studies for data on the complete composition of floral scents and the emission rates of each compound or alternatively describing the relative percentage of contribution of each compound to the blend. We aimed to identify the effects of biological (pollinators) and climatic factors on the floral scents of the species by combining the data on the floral scents with the available data on the pollinators and climatic conditions in the regions where the plant species were sampled.

We compiled the available information on floral-VOC emission rates and/or relative VOC compositions for 305 plant species from 66 families. The database we compiled contained >800 compounds classified into nine groups: fatty acid derivatives, amino acid derivatives, benzenoids, monoterpenes, sesquiterpenes, irregular terpenes, nitrogen-containing compounds, sulphur-containing compounds, and miscellaneous compounds. We also obtained information about the location and the climatic conditions where the populations from the different species that were measured grew and about the type of pollinators that visited the flowers, as described by the original studies. The information contained in our database allowed us to identify the most ubiquitous and dominant VOCs in floral scents (those that more frequently had the highest relative abundances in floral VOC blends). We further determined whether phylogeny, reliance on different types of pollinators, and climate were correlated with floral VOC richness, scent composition, and rate of emission. We hypothesized that the compositions and rates of emission of floral scents have been preserved throughout evolutionary history, and we aimed to differentiate between the effects of phylogeny on floral scents and the effects of biotic and climatic factors. We expected that the pollination syndrome would be correlated with VOC richness, composition, and emission rate of floral scents. Finally, we hypothesized that climate would exert some selective pressures on the production and emission of floral scents, thus positively or negatively stimulating the emissions of all or some compounds under the environmental conditions where each species grew and flowered.

## Methods

### Search Criteria

We exhaustively searched the Web of Knowledge and Google Scholar for studies of floral scent using combinations of the keywords “floral”/“flower” and “volatiles”/”VOCs”/“scent”. We chose studies that provided complete data on the emission rates and/or relative percentages of all floral VOCs emitted. We discarded studies that did not report the complete bouquet of VOCs of the floral scents but focused only on particular compounds, thus omitting other compounds that were emitted but were not the focus of the study. We finally selected 58 studies that provided information on the complete compositions of floral scents of one or more species. The references for all the studies from which we used data to make our database can be found in **Table S1**, where all plant species included in the database are found classified by families.

### Data Entry

Each case in our database corresponded to a description of the floral scent of one plant species in one study. The name of the species (and subspecies when appropriate) was entered as provided by the source study, and equivalent synonyms following the Angiosperm Phylogeny Group III classification system, the genera, and the families were also recorded in our database. We obtained the longitudes and latitudes of the populations from which individuals of each species were sampled according to the methods described in the papers, and several climatic variables were obtained from the *WorldClim* database: Mean Annual Temperature, Max Temperature of Warmest Month and Min Temperature of Coldest Month (K), Mean Annual Precipitation, Precipitation of Wettest Month, and Precipitation of Driest Month (L/m^2^). We calculated the Gaussen index of aridity using the climatic data as: *Gaussen index* = *annual precipitation*/(2 * mean annual temperature). We further obtained information on the pollinators that visited the flowers of each plant species as described by the source studies.

We classified floral VOCs based on their biosynthetic pathways, which was the predominant classification in all our data sources and enabled comparisons with phylogenetic hypotheses. We thus divided floral VOCs into the nine major classes: fatty acid derivatives, amino acid derivatives, benzenoids, monoterpenes, sesquiterpenes, irregular terpenes, nitrogen-containing compounds, sulphur-containing compounds, and miscellaneous compounds. We entered the data on the floral scent of each species as “presence/absence” (1/0), “relative percentages of the total blend” (%), and “emission rates” for all individual compounds and for the nine major groups of floral volatiles identified above. The source studies did not always provide emission rates, but all studies provided percentages of the total floral VOC blend. Emission rates were provided in various units (*μg h^-1^ flower^-1^*, *μg h^-1^ inflorescence^-1^*, *μg h^-1^ g DW^-1^*, and *μg h^-1^ g FW^-1^*), depending on the methods used in each study. Some studies specified the isomer(s) of various isomeric compounds in the floral scent of the species, but others did not. Several compounds thus appear in our database as repeated variables with and without a specified isomer.

### Classification of the Plant Species Into Pollination Syndromes

We obtained information from the source studies of the pollinators that visited each plant species. Several species were pollinated or visited by different pollinator groups, so we created several binary variables in our database to indicate whether a particular plant species was or was not pollinated by a particular pollinator group (*wind*, *animals*, *insects*, *Lepidoptera*, *Coleoptera*, *Diptera*, *Hymenoptera*, *bats*, and *birds*). The description of pollinators differed across classification levels amongst the studies, so we created different binary variables to characterize the spectrum of pollinators, some of which were included in other groups (e.g. *Lepidoptera*<*insects*<*animals*) and provided information that was redundant to some extent. Both higher and lower classification levels, however, were useful for conducting different comparisons to answer different questions.

We further classified as many plant species as possible (239 from a total of 305) as predominantly pollinated by *wind*, *Lepidoptera*, *Coleoptera*, *Hymenoptera*, *Diptera*, *bats*, or *birds*. Plant species for which no information on main pollinator type was provided or those that were generalists (pollinated by different pollinator groups) to important degrees could not be classified into these groups and were therefore not included in the classification figures or analyses that required this classification.

### Statistical and Phylogenetic Analyses

We prepared a phylogenetic tree containing the species in our database to test whether emission traits were phylogenetically preserved using R statistical software (R Core Team, 2017). We thereby obtained a phylogenetic tree containing a selection of 193 species from PhytoPhylo, an available megaphylogeny of vascular plants (Qian and Jin, 2016). We used the *phylosig* function from the R package *phytools* (Revell, 2012) to test for phylogenetic signals for floral VOC richness, composition, and emission rate for the species. The *phylosig* function calculates statistics of a phylogenetic signal (Pagel’s λ and Blomberg’s K) and *P* values based on the variance of phylogenetically independent contrasts relative to tip shuffling randomisation (Blomberg et al., 2003).

We also used the phylogenetic tree to reconstruct the ancestral states of floral VOC emissions and pollination syndromes. We used stochastic character mapping (Nielsen, 2002; Huelsenbeck et al., 2003) to reconstruct ancestral transitions amongst the emission types and the pollination syndromes across the phylogeny. This method reconstructs the state of the ancestors of a phylogeny based on its structure and the observed traits of the current species. The ancestral reconstructions were prepared using the *make.simmaps* function of the R package *phytools* (Revell, 2012), simulating 1,000 stochastic ancestral reconstructions using the “mcmc” method (Markov chain Monte Carlo) and specifying equal rates of transition amongst the character states. The trees were simulated with a discrete-character map, with the states representing the dominant groups of floral VOCs (*fatty acid derivatives*, *benzenoids*, or *terpenoids*) and the pollination syndromes (*wind*, *bats*, *birds*, *Coleoptera*, *Lepidoptera*, *Hymenoptera*, or *Diptera*).

We used the Kruskal-Wallis (K-W) test for non-parametric data to test for differences in floral VOC richness and in the percentages of the classes of volatiles between plants pollinated by different pollinator groups. We further used the K-W test to compare the percentages of the most common floral volatiles, i.e. benzaldehyde, limonene, linalool, trans-β-ocimene, and benzyl alcohol, in floral blends amongst plants pollinated by different pollinators. K-W tests were conducted with R software using the *kruskal* function of the *agricolae* package (De Mendiburu, 2009). We performed multiple comparisons with the same predictor (pollination syndrome), so we used Bonferroni correction for multiple comparisons (α = 0.05/*number of comparisons*).

We analyzed the effects of the pollinator types and climatic variables on VOC richness, relative percentages, and emission rates of each chemical class using phylogenetic linear regression models with R software. We used the *phylolm* function of the *phylolm* package, which fits phylogenetic linear models, allowing us to exclude the effect of phylogenetic distance (Ho and Ane, 2014). We tested for the effects of pollinator types using the binary variables describing whether the species were pollinated/visited by *wind*, *Lepidoptera*, *Coleoptera*, *Diptera*, *Hymenoptera*, *bats*, or *birds*. For emission rates, we conducted the phylogenetic linear regression models only with the data from species whose emissions were in units of *μg h^-1^ flower^-1^*.

We tested whether plant species that shared the main group of pollinators had similar compositions of floral-scent bouquets using non-metric multidimensional scaling (NMDS) based on two distance measures and then fitted the pollination system onto the ordination using the *envfit* function in the R package *vegan* (Dixon, 2003; Oksanen et al., 2018). We used Bray-Curtis distances implemented in *vegan* that considers each compound as an independent variable and measures the similarities in the percentages of emission of individual compounds. We also applied the biosynthetically informed distance measure, *d_A,B_* (Junker, 2018), that considers the shared biosynthesis of compounds. Each compound was assigned to one of the nine major classes of compounds described above. *d_A,B_* informs the proportion of shared biosynthetic pathways leading to the floral-scent emissions of the plant species. Finally, we merged Bray-Curtis and *d_A,B_* distances in different ratios using weight w to calculate the weighted mean of both distance measures (see Junker (2018) for details). These merged distances compensate for the lack of information of the enzymes involved in the biosynthesis of the compounds.

## Results and Discussion

### Diversity and Distribution of Floral Scent

We compiled 851 VOCs in the floral scents of 305 plant species belonging to 66 families (**Table S1**). Terpenoids were the most common floral volatiles (in the floral scents of 88.2% of the species), followed by benzenoids (80.7%), fatty acid derivatives (77.4%), nitrogen-containing compounds (30.8%), amino acid derivatives (9.2%), and sulphur-containing compounds (3.6%; **Table S2**). Phylogenetic signals were detected for floral VOC richness, relative composition and emission rates for some groups of compounds (**Table 1**), thus supporting that to some extent the compositions and rates of emission of floral scents have been preserved throughout evolutionary history.

**Table 1:** Results of the phylogenetic signal tests for richness (N = 197), relative percentage (N = 197), and emission rate (N = 67) of fatty acid derivatives (FADs), amino acid derivatives (AADs), benzenoids, monoterpenes, sesquiterpenes, irregular terpenes, terpenes, nitrogen-containing compounds (NCCs), and sulphur-containing compounds (SCCs).

	Richness				Relative percentage				Emission rate			
	λ	*P*	K	*P*	λ	*P*	K	*P*	λ	*P*	K	*P*
FADs	**0.40**	**0.015**	**0.08**	**<0.001**	0.42	0.05	**0.07**	**0.003**	**0.22**	**0.018**	0.07	0.37
AADs	**0.27**	**<0.001**	**0.08**	**0.04**	0.06	0.24	0.04	0.625	0.11	0.298	0.07	0.292
Benzenoids	**0.52**	**<0.001**	**0.05**	**0.022**	0.18	0.204	0.03	0.359	**0.47**	**0.012**	0.05	0.457
Monoterpenes	<0.01	1	0.03	0.351	**0.39**	**0.026**	0.03	0.102	**0.75**	**0.010**	0.07	0.427
Sesquiterpenes	0.04	0.538	0.03	0.4	<0.01	1	**0.13**	**0.004**	<0.01	1	0.04	0.594
Irr. terpenes	<0.01	1	**0.08**	**0.011**	0.8	1	**0.08**	**0.047**	**0.33**	**<0.001**	0.06	0.261
Terpenes	0.09	0.191	0.03	0.332	0.39	0.068	**0.04**	**0.025**	**0.75**	**0.008**	0.07	0.427
NCCs	**0.26**	**0.028**	0.02	0.781	0.14	0.753	0.02	0.697	<0.01	1	0.07	0.445
SCCs	**0.96**	**<0.001**	0.07	0.36	**0.97**	**<0.001**	0.19	0.151	<0.01	1	0.02	0.821

Pagel’s λ, Bloomberg’s K, and their associated P values are provided for each variable. Significant values are depicted in bold type.

Terpenoids, fatty acid derivatives, and benzenoids were the most diversified chemical groups of floral volatiles, with the highest richness of compounds. Terpenoids and benzenoids were the most predominant in the floral scents, followed by fatty acid derivatives (**Table S2**, **Figure S1**). These three groups of volatiles are the most important constituents of floral scents (Dobson, 2006). Other chemical groups were much rarer, sometimes only present or dominant in a small group of species. Sulphur-containing compounds were a special case; their occurrence and higher relative abundance in floral scents was strictly associated with bat pollination. These results are in accordance with findings that bat-pollinated plants attract pollinators by emitting floral scents rich in sulphur-containing compounds (Knudsen and Tollsten, 1995; Bestmann et al., 1997; von Helversen et al., 2000; Knudsen et al., 2006).

**Figure 1:**
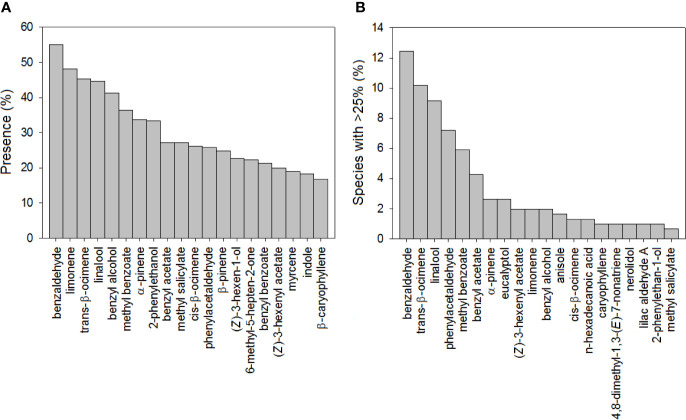
Bar chart showing **(A)** the percentage of plant species with the most common floral volatiles and **(B)** the percentage of plant species where the most abundant floral volatiles represented more than 25% of the total floral scent (N = 305).

Our database of floral VOC emissions identified the benzenoid benzaldehyde and the monoterpenes limonene, trans-β-ocimene, and linalool as the most ubiquitous volatiles in floral scents (**Figure 1A**), coinciding with previous studies by Knudsen et al. (1993; 2006). We further found that benzaldehyde, trans-β-ocimene, and linalool were the most common predominant floral VOCs (**Figure 1B**). This finding strongly supports the important ecological role in floral scents of β-ocimene, which is a common floral volatile emitted by plants pollinated by different groups of pollinators (Dobson, 2006; Knudsen et al., 2006; Filella et al., 2013; Farré-Armengol et al., 2017). Benzaldehyde and linalool are also good attractants of Lepidoptera (Dobson, 2006), which are a predominant group of pollinators of many angiosperms (Andersson et al., 2002; Dötterl et al., 2006). Linalool also has various other functions in floral ecology, ranging from repellent properties to effects in interactions with bacteria (Raguso and Pichersky, 1999a; Junker and Blüthgen, 2008; Raguso, 2016; Burdon et al., 2018).

### Pollination Syndromes and the Composition and Emission Rates of Floral Scents

Pollination syndromes consist of particular combinations of floral traits that attract particular groups of pollinators with shared floral preferences (Faegri and van der Pijl, 1979; Fenster et al., 2004). Plants pollinated by different pollination vectors are thus expected to emit floral VOC blends dominated by different types of compounds (Dobson, 2006). The plant species included in our floral-scent database represented the different main pollination vectors relatively well, although some of them were more represented than others, with Lepidoptera pollination the most frequent pollination vector in our data set (47.68%) (**Figure S2A**). We phylogenetically reconstructed the trait “main pollination vector” from the plants included in our database, which indicated how the main pollination vectors were distributed in the phylogeny and how the species switched from one pollination vector to another within the evolution of different plant lineages (**Figure S2B**). We also found that all the pollination vectors were distributed in different branches in the phylogeny, despite some phylogenetic clustering.

**Figure 2:**
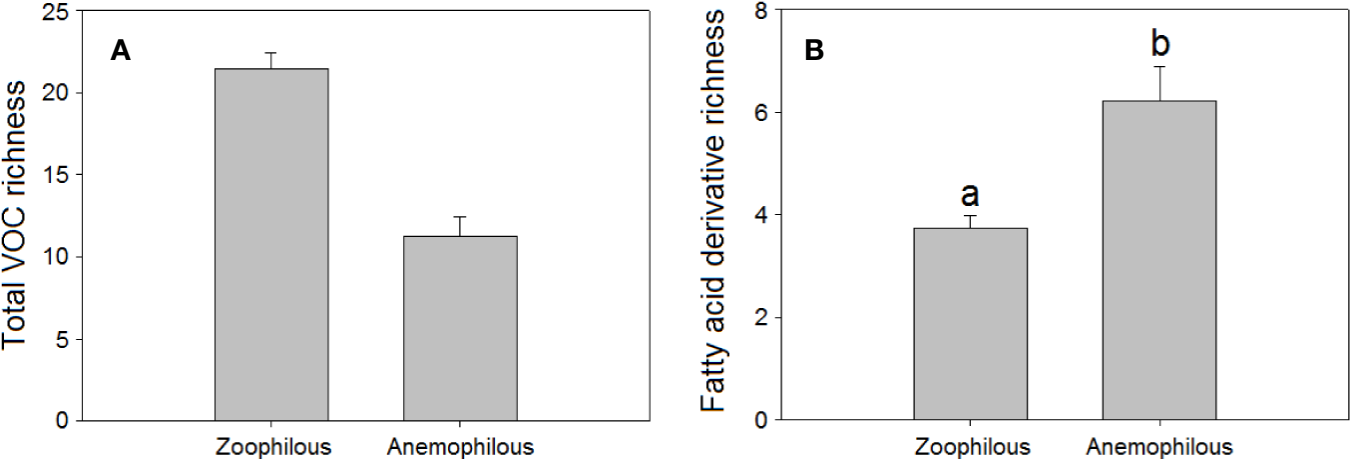
Richness of VOCs in the floral scents of zoophilous (N = 254) and anemophilous plant species (N = 9): **(A)** total VOC richness, **(B)** fatty acid derivative richness. Error bars indicate standard errors of the means. Different letters indicate significant differences between groups (Kruskal-Wallis test, α = 0.0056).

**Figure 3:**
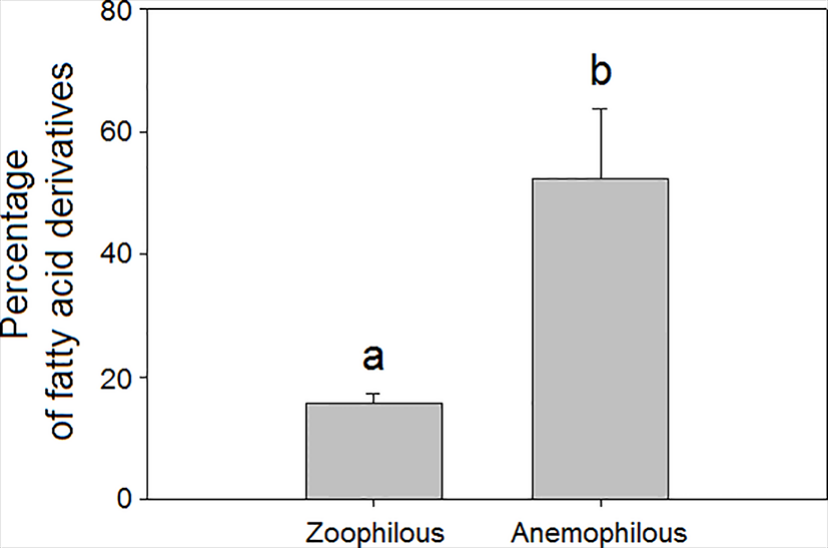
Relative percentage composition of VOCs in the floral scents of zoophilous (N = 9) and anemophilous plant species (N = 254): percentages of fatty acid derivatives. Error bars indicate standard errors of the means. Different letters indicate significant differences between groups (Kruskal-Wallis test, α = 0.0063).

We found that mean total floral VOC richness was higher in zoophilous species (pollinated by animals) as a group than in anemophilous species (pollinated by wind), although the differences were not significant (H = 4.06, *P* = 0.044, **Figure 2A**). Some studies have demonstrated that plants pollinated by wind tend to emit fewer floral volatiles and in lower amounts than do entomophilous plants (Magalhães et al., 2005; Wragg and Johnson, 2011; Farré-Armengol et al., 2015b). Zoophilous plants need to attract pollinators to their flowers to cross-pollinate them, using VOCs, visual signals, and floral rewards (Raguso, 2004; Whitehead and Peakall, 2009; Schiestl, 2010; Kantsa et al., 2017). Anemophilous plants, though, do not need to attract pollinators to their flowers to be pollinated and tend to emit weak floral scents, although they can emit some VOCs that may have functions other than pollinator attraction, such as defence. We found that anemophilous flowers emitted a significantly higher diversity (H = 8.75, *P* = 0.003, **Figure 2B**) and higher proportions of fatty acid derivatives than did entomophilous flowers (H = 13.7, *P* < 0.001, **Figure 3**). We hypothesize that VOC emissions of anemophilous flowers were dominated by fatty acid derivatives because some of the most common compounds in this group, the green leaf volatiles (GLV), develop defensive functions in vegetative as well as in other plant tissues (Scala et al., 2013; Naeem ul Hassan et al., 2015), and anemophilous plants are not negatively affected by presenting defensive (toxic or deterrent) compounds in their flowers, as zoophilous plants do (Lucas-Barbosa et al., 2011; Schiestl et al., 2011; Farré-Armengol et al., 2013).

**Figure 4:**
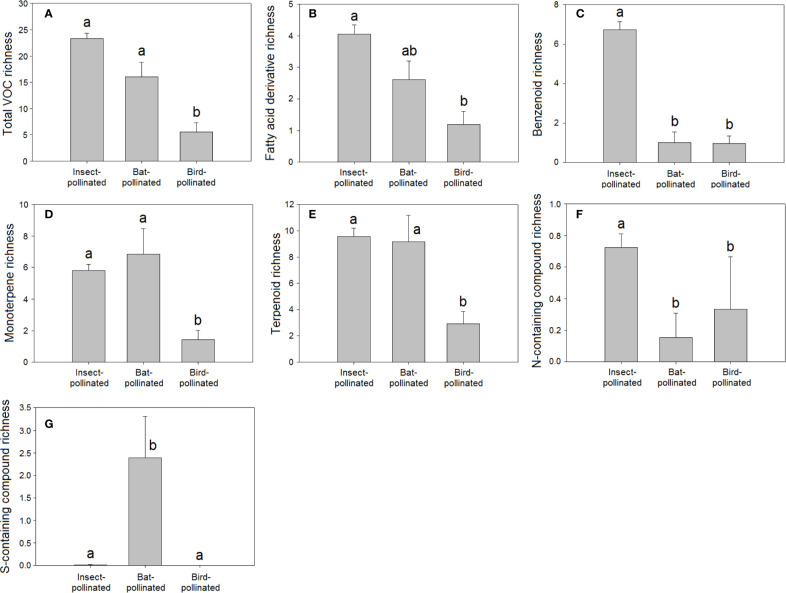
Richness of VOCs in the floral scents of plant species pollinated by insects (N = 221), birds (N = 21), and bats (N = 13): **(A)** total VOC richness, **(B)** fatty acid derivative richness, **(C)** benzenoid richness, **(D)** monoterpene richness, (**E)** terpenoid richness, **(F)** nitrogen-containing compound richness, and **(G)** sulphur-containing compound richness. Error bars indicate standard errors of the means. Different letters indicate significant differences between groups (Kruskal-Wallis test, α = 0.0056).

Ornithophilous flowers (pollinated by birds) are almost scentless (Knudsen et al., 2004; Magalhães et al., 2005; Klahre et al., 2011), likely because birds rely more on vision than olfaction for floral location (Faegri and van der Pijl, 1979; Knudsen et al., 2004; Cronk and Ojeda, 2008). The lower VOC richness in species pollinated by birds (H = 38.11, *P* < 0.001, **Figure 4A**) and the negative correlation detected between bird pollination and total VOC richness (**Table 2**) supported this proposal.

**Table 2:** Significant results of the phylogenetic linear models (*phylolm*) testing for the effects of pollination vectors and climatic variables on the richness of total VOCs, fatty acid derivatives (FADs), amino acid derivatives (AADs), benzenoids, terpenoids, monoterpenes, sesquiterpenes, nitrogen-containing compounds (NCCs), and sulphur-containing compounds (SCCs) (N = 142).

	β	*P*
Total VOCs vs. Lepidoptera pollination	0.341	<0.001
Total VOCs vs. Bird Pollination	-0.24	0.036
FADs vs. Mean annual precipitation	13.763	0.014
FADs vs. Gaussen index	-13.347	0.016
Benzenoids vs. Lepidoptera pollination	0.253	0.009
Benzenoids vs. Bird pollination	-0.316	0.003
Benzenoids vs. Maximum temperature of warmest month	-0.617	0.045
Terpenoids vs. Lepidoptera pollination	0.236	0.023
Monoterpenes vs. Bat pollination	0.265	0.003
Sesquiterpenes vs. Lepidoptera pollination	0.228	0.033
Sesquiterpenes vs. Mean annual temperature	1.849	0.004
Sesquiterpenes vs. Maximum temperature of warmest month	-0.922	0.004
Sesquiterpenes vs. Minimum temperature of coldest month	-1.303	0.006
NCCs vs. Lepidoptera pollination	0.488	<0.001
NCCs vs. Coleoptera pollination	0.319	0.003
NCCs vs. Diptera pollination	0.245	0.004
NCCs vs. Hymenoptera pollination	0.231	0.017
NCCs vs. Bird pollination	0.33	0.004
SCCs vs. Bat pollination	0.514	<0.001
SCCs vs. Precipitation of wettest month	-1.073	<0.001
SCCs vs. Precipitation of driest month	-0.779	<0.001

The function phylolm fits phylogenetic linear models that allow the exclusion of the effect of phylogenetic distance. The standardized coefficients and P values are provided for each variable.

**Figure 5:**
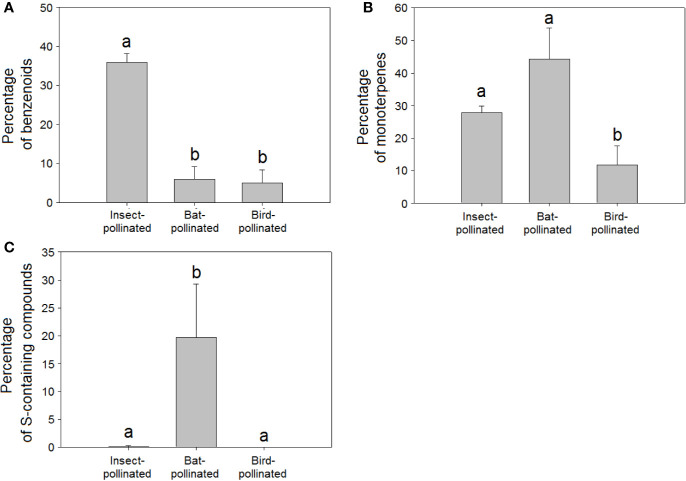
Relative percentage composition of VOCs in the floral scents of plant species pollinated by insects (N = 221), birds (N = 21), and bats (N = 13): percentages of **(A)** benzenoids, **(B)** monoterpenes, **(C)** sulphur-containing compounds. Error bars indicate standard errors of the means. Different letters indicate significant differences between groups (Kruskal-Wallis test, α = 0.0063).

The higher richness (H = 91.95, *P* < 0.001, **Figure 4G**) and relative percentage of sulphur-containing compounds in floral scents from bat-pollinated plants (H = 91.73, *P* < 0.001, **Figure 5C**) and the strongly significant positive correlations of bat pollination with both sulphur-containing compound richness (**Table 2**) and relative percentage (**Table 3**) supported a close relationship between the emission of sulphur-containing compounds and pollination mediated by bats. These results were in agreement with studies demonstrating convergent evolution of bat-pollinated plant species from different families to emit sulphur-containing volatiles such as dimethyl disulphide, dimethyl trisulphide, and dimethyl tetrasulphide (Knudsen and Tollsten, 1995; Bestmann et al., 1997). However, this pattern is not universal and seems to be restricted to bat-pollinated plants from the neotropics (Carter and Stewart, 2015). The emission of sulphur-containing compounds by neotropical bat-pollinated plants is an adaptation to attract flower-visiting bats that share an innate preference for this group of volatiles (von Helversen et al., 2000).

**Table 3:** Significant results of the phylogenetic linear models (*phylolm*) testing for the effects of pollination vectors and climatic variables on the relative percentage of fatty acid derivatives (FADs), amino acid derivatives (AADs), benzenoids, terpenoids, monoterpenes, sesquiterpenes, nitrogen-containing compounds (NCCs), and sulphur-containing compounds (SCCs) (N = 142).

	β	*P*
FADs vs. Wind pollination	0.289	0.001
FADs vs. Mean annual precipitation	0.194	<0.001
FADs vs. Gaussen index	-0.184	<0,001
Benzenoids vs. Bird pollination	-0.338	0.004
Terpenoids vs. Minimum temperature of coldest month	0.929	0.042
Terpenoids vs. Mean annual precipitation	-0.125	0.025
Terpenoids vs. Gaussen index	0.114	0.038
Monoterpenes vs. Mean annual temperature	-1.717	0.003
Monoterpenes vs. Maximum temperature of warmest month	1.081	<0.001
Monoterpenes vs. Minimum temperature of coldest month	1.844	<0,001
Monoterpenes vs. Mean annual precipitation	-0.148	0.005
Monoterpenes vs. Gaussen index	0.137	0.008
Sesquiterpenes vs. Mean annual temperature	1.4	0.03
Sesquiterpenes vs. Minimum temperature of coldest month	-0.966	0.042
SCCs vs. Bat pollination	0.402	<0.001
SCCs vs. Precipitation of wettest month	-1.824	<0.001
SCCs vs. Precipitation of driest month	-1.098	<0.001

The function phylolm fits phylogenetic linear models that allow the exclusion of the effect of phylogenetic distance. The standardized coefficients and P values are provided for each variable.

**Figure 6:**
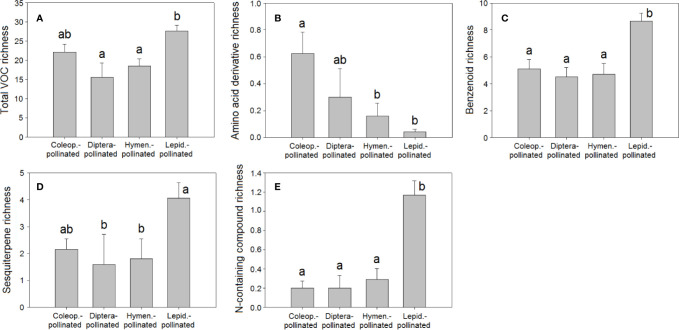
Richness of VOCs in the floral scents of plant species pollinated by Coleoptera (N = 40), Diptera (N = 10), Hymenoptera (N = 31), and Lepidoptera (N = 113): **(A)** total VOC richness, **(B)** amino acid derivative richness, **(C)** benzenoid richness, **(D)** sesquiterpene richness, **(E)** nitrogen-containing compound richness. Error bars indicate standard errors of the means. Different letters indicate significant differences between groups (Kruskal-Wallis test, α = 0.0056).

**Figure 7:**
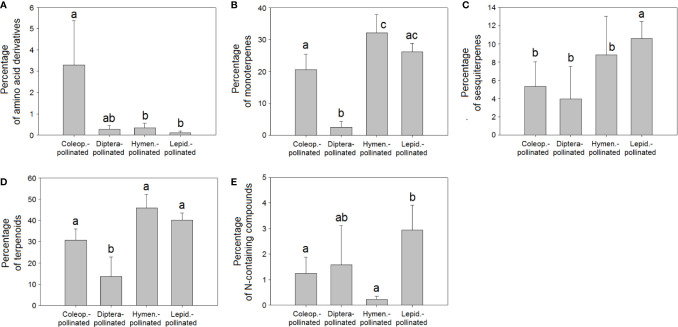
Relative percentage composition of VOCs in the floral scents of plant species pollinated by Coleoptera (N = 40), Diptera (N = 10), Hymenoptera (N = 31), and Lepidoptera (N = 113): percentages of **(A)** amino acid derivatives, **(B)** monoterpenes, **(C)** sesquiterpenes, **(D)** terpenoids, **(E)** nitrogen-containing compounds. Error bars indicate standard errors of the means. Different letters indicate significant differences between groups (Kruskal-Wallis test, α = 0.0063).

The differences in floral VOC richness (**Figures 4** and **6**), floral-scent composition (**Figures 5** and **7**), and relative abundance of individual compounds (**Figure S3**) amongst plant species pollinated by different animal groups may also support the existence of different pollination syndromes for floral scent. The higher richness of benzenoids and the monoterpene linalool in the scent of flowers pollinated by butterflies and moths strongly suggest a preference of Lepidoptera for these compounds (Raguso and Pichersky, 1999b; Andersson et al., 2002; Dötterl et al., 2006). Our results supported this preference: benzenoids were more diversified in the floral scents of Lepidoptera-pollinated plants (**Figure 6C**; **Table 2**), and linalool was also more dominant in Lepidoptera-pollinated species (**Figure S3C**). On the other hand, benzaldehyde is a common floral volatile that has been measured in important proportions in the floral scents of some plant species pollinated mainly by Diptera, such as *Leontopodium alpinum*, *Crataegus* sp., and *Filipendula ulmaria* (Dobson, 2006). Our results confirm that benzaldehyde was more abundantly represented in the floral scents of plant species that are pollinated by Diptera (**Figure S3A**). On the contrary, β-ocimene was recognized to be widely distributed in the floral scents of plants that belong to different pollination syndromes and has been proposed to play a key role as a generalist pollinator attractant (Filella et al., 2013; Farré-Armengol et al., 2017). Actually, trans-β-ocimene is a ubiquitous floral-scent constituent with high levels of occurrence and high relative abundances in floral scents (**Figure 1**) (Dobson, 2006; Knudsen et al., 2006), which usually co-occurs with its less abundant isomer, cis-β-ocimene (**Table S2**).

**Figure 8:**
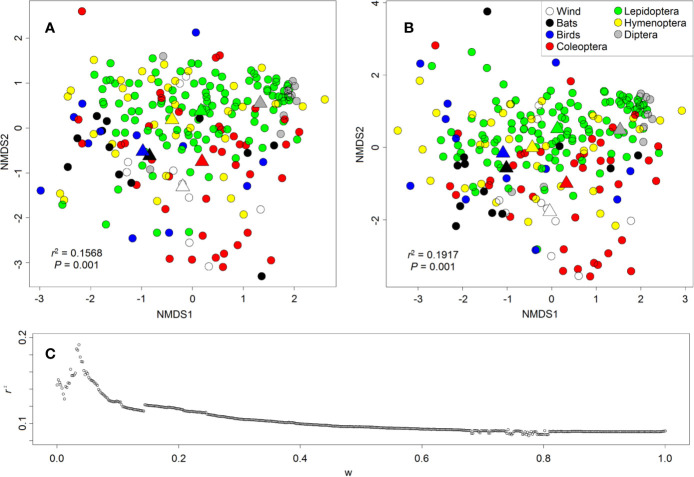
Ordination (NMDS) of floral-scent bouquets based on **(A)** Bray-Curtis distances and **(B)** biosynthetically informed distances *d_A,B_* merged with Bray-Curtis distances in a ratio 36:964. Main pollinators are colour-coded as shown in the legend in **(B)**. Each circle represents a plant species, and triangles are the centroids of scent bouquets of flowers pollinated by the same pollinators. Although no clear clusters of pollination systems are visible, the mean position of pollination systems in the ordination are still significantly different from each other (see centroids). **(C)** *r^2^* of fitting of pollination systems onto the ordination as a function of weight *w* to calculate merged distances (*0, Bray-Curtis distance; 1, biosynthetically informed distance measure d_A,B_*).

We found some support for the pollination syndromes in the composition of scent bouquets. The main pollinators significantly fitted onto the ordinations representing similarities in floral-scent composition between species, but no clear clusters were detected (**Figure 8**). Fitting the main pollinators onto the ordination resulted in lower *r^2^* values when NMDS was based on Bray-Curtis distances (*r*^2^ = 0.1568, *P* = 0.001, mean of 1,000 permutations, **Figure 8A**) than on biosynthetically informed distances *d_A,B_* merged with Bray-Curtis distances in a ratio 36:964 (*r*^2^ = 0.1917, *P* = 0.001, **Figure 8B**) (see Junker, 2018 for information on methodological details). Bray-Curtis distances consider each compound individually, whereas biosynthetically informed distances consider the proportion of shared major classes of compounds and therefore the proportion of shared biosynthetic pathways. This result indicates that often not specific compounds mediated the attraction of the main pollinators, but the presence/absence or abundance of compounds sharing the same biochemical pathway. Several case studies have highlighted the importance of key compounds in flower–pollinator interactions (Riffell et al., 2009; Svensson et al., 2010; Schäffler et al., 2015; summarized in Junker, 2016); our results suggest that compounds from the same biosynthetical pathway may have redundant functions—at least in the context of higher taxonomic levels as in pollination syndromes.

Floral-scent bouquets are accordingly less integrated than bouquets emitted by leaves, so the proportional composition of floral-scent bouquets is much more variable than that of foliar volatiles (Junker et al., 2017). Individual compounds (or representatives of chemical classes) may thus be sufficient to mediate functions such as pollinator attraction, regardless of the presence/absence or emission rate of other compounds in the bouquet (Junker et al., 2017). These findings in combination with the finding that some compounds are over-represented in some of the pollination systems (see above) support the concept that key compounds mediate interactions of flowers with their pollinators, not ratios of compounds or entire compound classes (Junker, 2016; Junker et al., 2017). Our results, combined with earlier findings, thus suggest that pollination syndromes that consider floral-scent emissions should not be defined based on the composition of the bouquets. The presence of individual key compounds or the presence of compounds originating from specific biosynthetical pathways may instead be indicative of pollination by a pollinator taxon. Note, however, that several compounds are over-proportionally found in the scent bouquets of plants pollinated by different taxa (the present study; Dobson, 2006), questioning the universal validity of these findings. The current data on floral-scent emission are generally strongly biased towards specialized plant-pollinator systems, and thus towards plant species that can be clearly assigned to a syndrome, which is also evident in our data set. Most plant species are visited and pollinated by several taxa, preventing the assignment of a plant species to a syndrome (Waser et al., 1996). Considering all pollinator assemblages and assessing the relative efficiency of all floral visitors are thus important for a better understanding of the role of plant-pollinator interactions in floral-trait evolution.

Secondary pollinators can play an important role in plant reproduction and floral-trait selection, potentially shifting evolutionary trends in pollination syndromes (Rosas-Guerrero et al., 2014). Plant fitness can significantly benefit from visits by pollinators that do not belong to the main functional group of pollinators (Fishbein and Venable, 1996; Miyake and Yahara, 1998; Kandori, 2002; Sahli and Conner, 2007). The suitability of attracting secondary or occasional pollinators to flowers can therefore also exert important selection pressures on floral traits (Aigner, 2001). Ollerton et al. (2009) proposed that selecting only the most effective pollinator failed to identify the range of logical possibilities that could account for the evolution of a floral trait. Some authors have suggested that the large temporal and spatial variation in the spectra of pollinators and effectiveness across years and locations may mitigate or dilute the relative impact of any specific pollinator as a selective agent on heritable floral variation (Herrera, 1996; Ollerton, 1996; Waser et al., 1996; Raguso, 2008b). This variation may also favour generalised reproductive strategies and phenotypes that attract multiple pollinators. Future research should therefore focus on plant species that are not involved in specialized pollination mutualisms but are visited by many taxa. Studying scent bouquets in plant communities may help us to find more universal patterns (Junker, 2016; Larue et al., 2016; Junker et al., 2017; Kantsa et al., 2017; Kantsa et al., 2018).

Other biological agents not yet mentioned, such as herbivores, floral larcenists, and other floral visitors with negative impacts on plant fitness, and floral microbial communities and pathogens may also have important effects on floral-trait evolution, including floral scent (Strauss and Armbruster, 1997; Frey, 2004; Lau and Galloway, 2004; Parachnowitsch and Caruso, 2008; Junker et al., 2011; Junker, 2016). Scent blends are generally composed of many volatiles, so different components of the blend may play different roles and be under different forms of selection (Kessler et al., 2008; Schiestl et al., 2011). All these multiple agents of selection can exert different or even opposite selection pressures on the same floral traits (Cariveau et al., 2004; Kessler and Halitschke, 2009) and can have varying impacts across time and space (Brody, 1997; Kandori, 2002; Dupont et al., 2009; Schlumpberger et al., 2009).

### Links Between Climatic Variables and Floral Scent

We found several significant relationships between climatic variables and the richness, relative composition, and emission rate of floral VOCs (**Tables 2**–**4**). Richness and relative percentage of fatty acid derivatives in the floral scents were positively correlated with annual precipitation (**Tables 2** and **3**). The relative percentage of monoterpenes showed negative correlations with mean annual temperature and also with annual precipitation (**Table 3**); the later would indicate a prevalence of monoterpenes in drier conditions, which could link with the fact that monoterpene emissions, rather than sesquiterpene emissions, seem to protect plants against oxidative stresses throughout drought periods (Ormeño et al., 2007) and their emissions are enhanced under moderate drought stress (Vallat et al., 2005; Yani et al., 2006; Ormeño et al., 2007). The richness, relative percentage, and emission rates of sesquiterpenes were positively correlated with mean annual temperature (**Tables 2**–**4**); these results strongly support the observations from previous studies that indicated that sesquiterpene emissions from vegetation are dominated mainly by ambient temperature, with a positive effect of temperature on them (reviewed by Duhl et al., 2008). The emission rates of nitrogen-containing compounds were negatively correlated with mean annual precipitation (**Table 4**). All these results suggest that climate is a relevant factor determining the compositions and emission rates of floral scents, in addition to the strong and well-known selective pressures exerted by biotic agents such as pollinators and other floral visitors (Jacobsen and Olsen, 1994; Yua et al., 2009; Farré-Armengol et al., 2013). The maximum temperatures that plant species can experience in their region during flowering, for example, have been positively correlated with the species-specific temperature thresholds that decrease floral-scent emissions, i.e. the maximum temperature tolerance of floral-scent emissions (Farré-Armengol et al., 2015a). Plants can thus adapt their physiology to optimise floral-scent emissions under the climatic conditions where they grow and flower. The responses of VOC emissions under particular environmental conditions are determined not only by plant physiology, but also by the temperature responses of the enzymes involved in their biosynthesis, the temperature responses of the membrane transporters and the cuticle composition and thickness, and also the particular physicochemical properties of the compounds (Niinemets et al., 2004; Copolovici and Niinemets, 2005; Noe et al., 2006; Harley, 2013; Farré-Armengol et al., 2014). We therefore hypothesize that climate can also select for floral scents that contain compounds with different physicochemical properties and increase the suitability of floral scents to the environmental conditions that plants and their flowers experience.

**Table 4:** Significant results of the phylogenetic linear models (*phylolm*) testing for the effects of pollination vectors and climatic variables on the emission rates of total VOCs, fatty acid derivatives (FADs), amino acid derivatives (AADs), benzenoids, terpenoids, monoterpenes, sesquiterpenes, nitrogen-containing compounds (NCCs), and sulphur-containing compounds (SCCs) (N = 43).

	Estimate	*P*
Sesquiterpenes vs. Mean annual temperature	3.804	0.006
Sesquiterpenes vs. Maximum temperature of warmest month	-1.621	0.012
Sesquiterpenes vs. Minimum temperature of coldest month	-3.038	0.002
Sesquiterpenes vs. Precipitation of wettest month	3.368	0.006
NCCs vs. Maximum temperature of warmest month	1.622	0.027
NCCs vs. Mean annual precipitation	-0.289	0.024
NCCs vs. Gaussen index	0.306	0.022

The function phylolm fits phylogenetic linear models that allow the exclusion of the effect of phylogenetic distance. The standardized coefficients and P values are provided for each variable.

### Future Prospects

Research bias from the non-random sampling of the natural world is an important problem in any review (Gurevitch and Hedges, 1999), and the authors cannot correct for it. Identifying gaps in the literature where more research is needed, however, is an important contribution of any review. The available information on floral scents that we compiled was collected for many families representing a broad phylogenetic range, which allowed us to characterize general trends in the distribution of floral emissions. The species also belonged to different pollination syndromes and had different geographical distributions, which allowed us to explore the relationships between floral scents and biotic and climatic factors. The floral scents for some pollination syndromes, however, are poorly represented in our database, especially those from plant species pollinated by wind, birds, bats, and Diptera. Most angiosperms rely on animals for pollination, and fewer species rely on abiotic vectors such as wind (Ollerton et al., 2011). Floral scents are especially associated with biotic pollination, so ecological studies of floral-scent chemistry tend to focus on the floral scents of animal-pollinated plants and their role in the attraction of the pollinators. The smaller proportion of wind-pollinated species, and the smaller biological and ecological interest in characterizing their floral scents, may have therefore strongly biased what we know about the floral scents of wind-pollinated plants compared to the floral scents from other pollination syndromes. The same is true for bird-pollinated plant species. Studies of the pollination ecology of bird-pollinated species often focus on visual and morphological floral traits rather than scent, because birds rely more on vision than olfaction for floral location (Cronk and Ojeda, 2008), and bird-pollinated species emit weak or no floral scents (Knudsen et al., 2004; Magalhães et al., 2005).

The most important gap in our knowledge of floral scents is probably the scents of plant species with generalist spectra of pollinators. Species with generalist spectra of pollinators are under-represented in the floral-scent literature, because most studies of floral scent have focused on species with specific pollinator interactions. However, not all floral visitors are effective pollinators, and only some generate selection on plant and floral traits, despite receiving visits by two or more groups of floral visitors (Armbruster et al., 2000). We consider that this fact is important for our analyses, something that could be improved if the studies provided more accurate and detailed data and a greater certainty identifying the effective pollinators. Most studies analysing and describing the floral scents of animal-pollinated plant species that are not strict specialists unfortunately do not describe the relative importance of their pollinators in much detail. We thus focused on plants that most clearly belonged to the “specialist” syndromes when analysing the pollination syndromes. New studies of floral-scent biology and chemistry should continue to expand our current knowledge of the distribution of floral scents, taking special care to also characterize the less well represented groups of plants, including species from all families, pollination syndromes, and climatic regions.

Some studies of the composition of floral scents provided emission rates for all floral compounds, but many studies provided only the relative percentages. The relative composition of floral scents is very relevant information, but actual emission rates could contribute more to our understanding of floral scents. We therefore encourage authors to quantify and describe the emission rates for each compound when possible. We also encourage authors to use the same reference units when providing emission rates, which would simplify the inclusion of their results in combined analyses. We noticed that μg h^-1^ flower^-1^ was the most commonly used unit of emission rate. We strongly recommend, however, the use of μmol h^-1^ g DW^-1^ (instead of, or in addition to, any other units), because it is a more standardized unit for describing emissions from flowers or any other plant organs/tissues.

The responses of floral scents to different environmental climatic factors such as temperature or drought are highly plastic (Farré-Armengol et al., 2013; Farré-Armengol et al., 2014; Glenny et al., 2018), as are the responses to biotic interactions (Huber et al., 2005; Lucas-Barbosa et al., 2011; Schiestl et al., 2011; Schiestl et al., 2014; Junker, 2016; Hoffmeister and Junker, 2017). The high plasticity of floral-scent emissions within individual plants is usually not considered in sufficient detail (Majetic et al., 2009). The current literature, however, highlights the great potential of analyzing intraspecific floral-scent variation, which occurs within and amongst populations and within individuals (Delle-Vedove et al., 2017).

Phyllospheric microorganisms have important effects on the composition of floral volatile emissions (Peñuelas et al., 2014; Helletsgruber et al., 2017). Microorganisms living on flowers can produce and emit VOCs, transform or degrade the VOCs emitted by floral tissues, and affect plant physiology, causing multiple changes to floral emissions (Junker and Tholl, 2013; Farré-Armengol et al., 2016a). Future research will verify the importance of microorganisms for defining the chemical phenotype of flowers and the implications for the biological interactions that these olfactory signals mediate.

## Conclusions

Floral scents are subject to many evolutionary pressures exerted by biotic and abiotic environmental factors. The need to attract pollinators is a major reason why animal-pollinated angiosperms have evolved complex and diverse floral scents. Plants have thus evolved various sets and mixtures of floral volatiles that help promote flower constancy, which in some cases stimulate the attraction of specific groups of pollinators. We identified some patterns indicating that particular VOCs were associated with particular pollination syndromes. Our results support the concept that key compounds or compounds originating from specific biosynthetic pathways play a significant role in mediating the interactions of flowers with their pollinators. Other floral visitors (e.g. herbivores and larcenists) and floral inhabitants (e.g. nectar yeasts and floral microbial communities and pathogens) also exert important selection pressures on plant secondary metabolism and floral scents. All these selection pressures act in different ways on floral phenotypes and they may difficult the appearance of patterns established across the entire phylogeny of flowering plants (or even within major plant clades) of shared emissions of complex VOC mixtures associated with the attraction of a particular pollinator group. We identified several significant relationships between climatic variables and the richness, relative composition, and emission rate of floral VOCs. Our results suggest that climate is a relevant factor determining the composition and emission rate of floral scents, in addition to the strong and well-known selective pressures exerted by biotic agents such as pollinators and other floral visitors. We therefore hypothesize that climate can also select for floral scents that contain compounds with different physicochemical properties, increasing the suitability of floral scents to the environmental conditions that plants experience during flowering.

## Data Availability Statement

All datasets presented in this study are included in the article/**Supplementary Material**.

## Author Contributions

GF-A and JP conceived and designed the study. GF-A compiled all the information contained in the database used in the study. MF-M and RJ significantly contributed to the statistical analysis of data by conducting some of the analyses and advising GF-A on the suitability of all the analyses employed. GF-A wrote the manuscript, and JP, RJ, MF-M, and IF supervised and contributed to the writing. All authors contributed to the article and approved the submitted version.

## Funding

This research was supported by the Austrian Science Fund (FWF, M2182), the Spanish Government grant CGL2016-79835, the Catalan Government grant SGR 2017-1005, the European Research Council Synergy grant ERC-2013-SyG-610028 IMBALANCE-P and the Catalan Government grant FI-2013. MF-M is a postdoctoral fellow of the Research Foundation – Flanders (FWO). The authors declare no conflicts of interest.

## Conflict of Interest

The authors declare that the research was conducted in the absence of any commercial or financial relationships that could be construed as a potential conflict of interest.

The reviewer [JM] declared a past co-authorship with one of the authors [RJ] to the handling Editor.

## Supplementary Material

Figure S1 Phylogenetic tree showing the dominant class of floral VOCs for the species included in the phylogeny of reference *PhytoPhylo* (small circles; N = 197) and the probability of emission type of ancestor nodes (large circles) as pie charts. The ancestral reconstruction was performed using 1,000 stochastic character-mapped trees (see the Methods section for further information).

Figure S2 **(A)** Pie chart showing the number of species in our data set that were pollinated by bats, birds, Coleoptera, Diptera, Hymenoptera, Lepidoptera, and wind (and in brackets the proportions they represent relative to the total number of N = 239 species that could be classified into the main pollination vector groups). **(B)** Phylogenetic tree showing the main pollination vector of the species included in the phylogeny of reference *PhytoPhylo* (small circles; N = 152) and the probability of main pollination vector types of ancestor nodes (large circles) as pie charts. The ancestral reconstruction was performed using 1,000 stochastic character-mapped trees (see the Methods section for further information).

Figure S3 Relative percentages of abundance of the most common floral volatiles in the floral scents of plants pollinated by bats (N = 12), birds (N = 21), Coleoptera (N = 37), Diptera (N = 9), Hymenoptera (N = 37), Lepidoptera (N = 114) and wind (N = 9): **(A)** benzaldehyde, **(B)** limonene, **(C)** linalool, **(D)** trans-β-ocimene, and **(E)** benzyl alcohol. Error bars indicate standard errors of the means. Different letters indicate significant differences between groups (Kruskal-Wallis test, α = 0.01).
