# Supplementary figures and images for: Deciphering the Biotic and Climatic Factors That Influence Floral Scents: A Systematic Review of Floral Volatile Emissions
Source: Front Plant Sci. 2020 Jul 31;11:1154. doi: 10.3389/fpls.2020.01154 (PMC7412988; doi:10.3389/fpls.2020.01154)

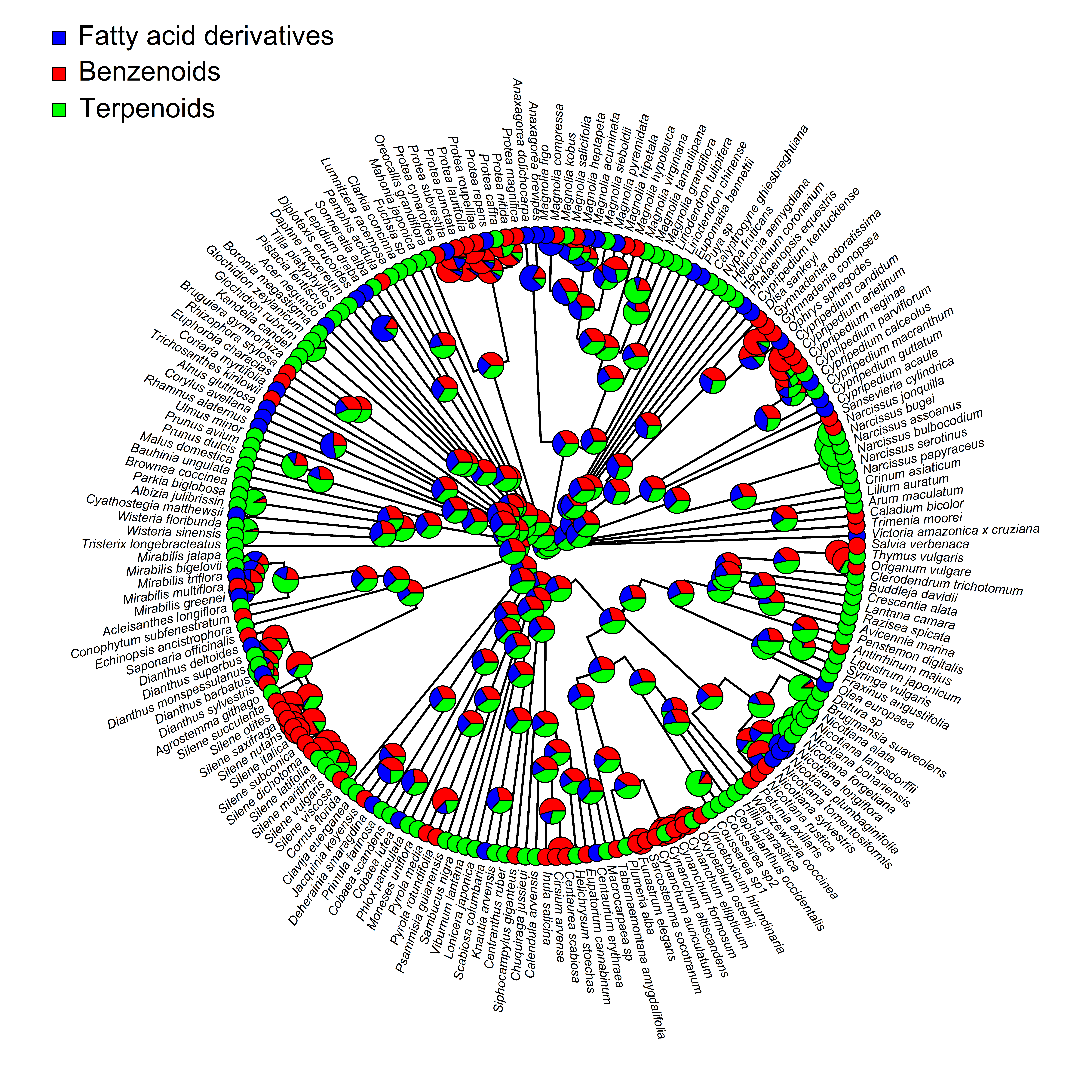

Supplement: Figure S1 — Phylogenetic tree showing the dominant class of floral VOCs for the species included in the phylogeny of reference PhytoPhylo (small circles; N = 197) and the probability of emission type of ancestor nodes (large circles) as pie charts. The ancestral reconstruction was performed using 1,000 stochastic character-mapped trees (see the Methods section for further information). [file Image_1.tiff]

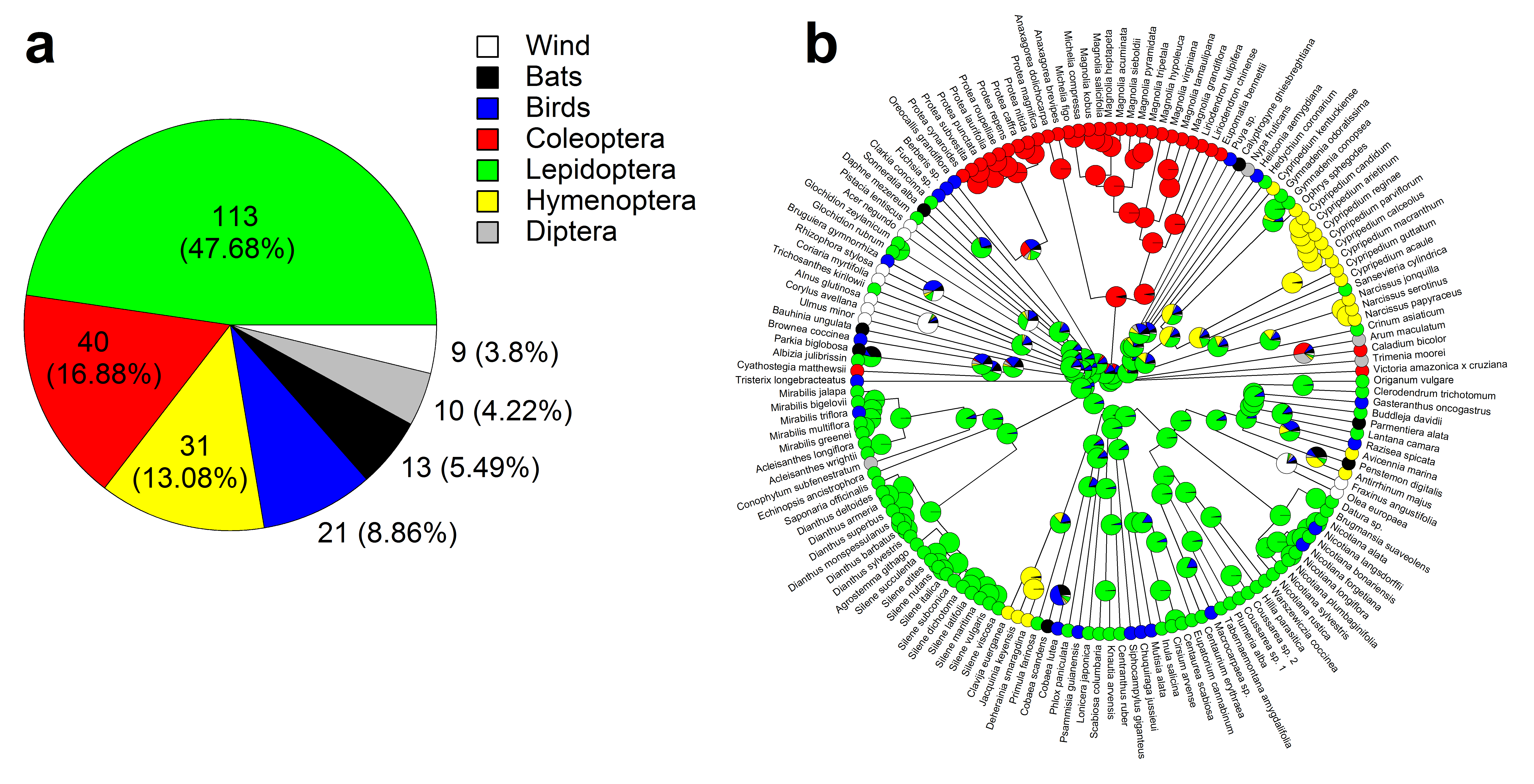

Supplement: Figure S2 — (A) Pie chart showing the number of species in our data set that were pollinated by bats, birds, Coleoptera, Diptera, Hymenoptera, Lepidoptera, and wind (and in brackets the proportions they represent relative to the total number of N = 239 species that could be classified into the main pollination vector groups). (B) Phylogenetic tree showing the main pollination vector of the species included in the phylogeny of reference PhytoPhylo (small circles; N = 152) and the probability of main pollination vector types of ancestor nodes (large circles) as pie charts. The ancestral reconstruction was performed using 1,000 stochastic character-mapped trees (see the Methods section for further information). [file Image_2.tiff]

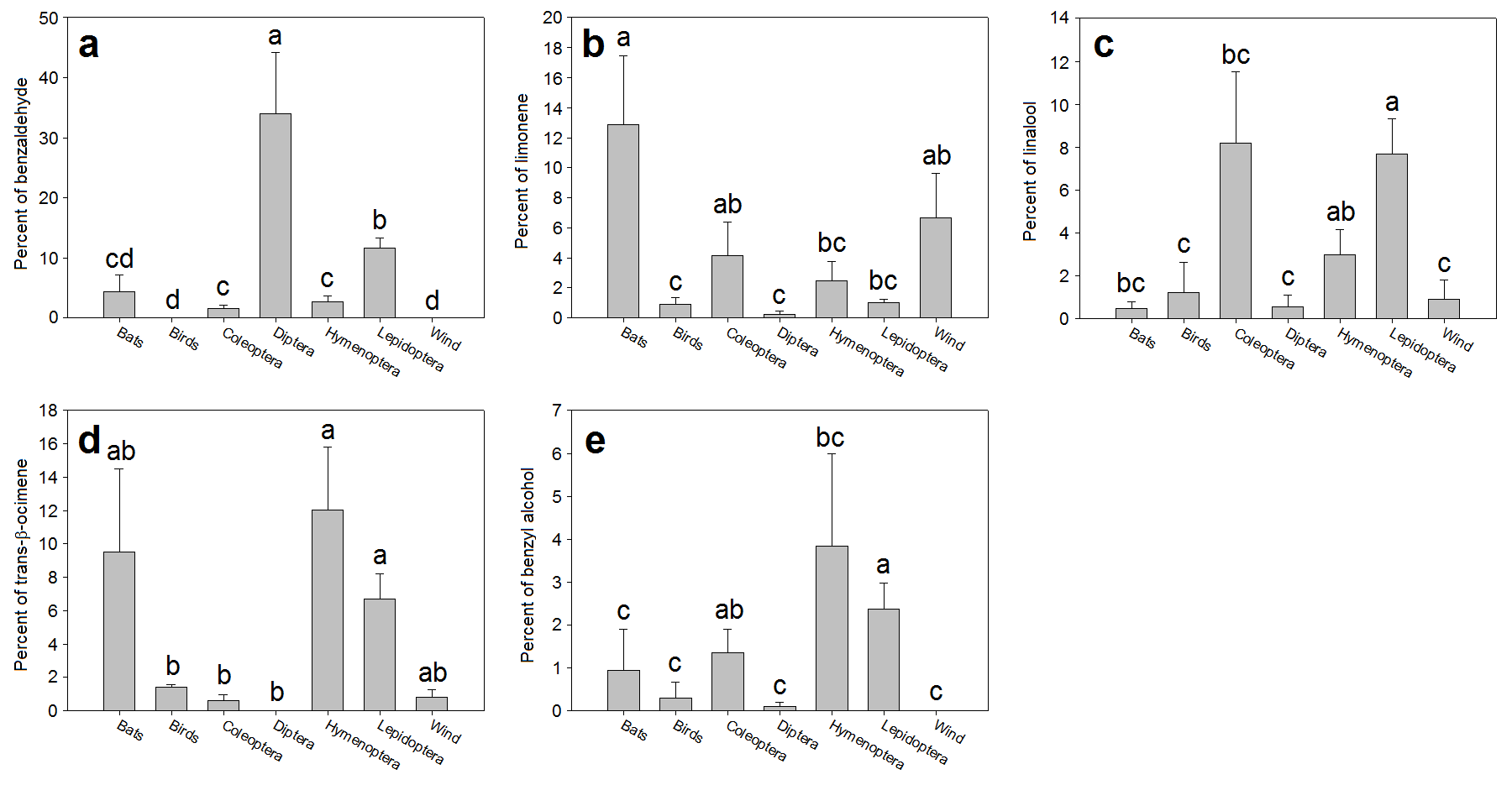

Supplement: Figure S3 — Relative percentages of abundance of the most common floral volatiles in the floral scents of plants pollinated by bats (N = 12), birds (N = 21), Coleoptera (N = 37), Diptera (N = 9), Hymenoptera (N = 37), Lepidoptera (N = 114) and wind (N = 9): (A) benzaldehyde, (B) limonene, (C) linalool, (D) trans-β-ocimene, and (E) benzyl alcohol. Error bars indicate standard errors of the means. Different letters indicate significant differences between groups (Kruskal-Wallis test, α = 0.01). [file Image_3.tif]
