# Supplementary material for: Deciphering the Biotic and Climatic Factors That Influence Floral Scents: A Systematic Review of Floral Volatile Emissions
Source: Front Plant Sci. 2020 Jul 31;11:1154. doi: 10.3389/fpls.2020.01154 (PMC7412988; doi:10.3389/fpls.2020.01154)
Supplement: Supplementary file 7 [file Table_1.docx]

**Table S1. List of plant species included in the database.** Superindices indicate the reference to the paper from which we obtained data on the floral emissions of each species: ^1^(Azuma et al., 2002); ^2^(Knudsen et al., 2004); ^3^(Farré-Armengol et al., 2015); ^4^(Jürgens, 2004); ^5^(Miyake et al., 1998); ^6^(Bestmann, 1997); ^7^(Dobson et al., 1997); ^8^(Jürgens et al., 2000); ^9^(Jürgens et al., 2009); ^10^(Knudsen and Tollsten, 1993); ^11^(Kuanprasert et al., 1998); ^12^(Hentrich et al., 2010); ^13^(Kite, 1995); ^14^(Maia et al., 2013); ^15^(Knudsen, 2002); ^16^(Andersson et al., 2002); ^17^(Kite and Smith, 1997); ^18^(Picone et al., 2002); ^19^(Schlumpberger and Raguso, 2008); ^20^(Jürgens et al., 2003); ^21^(Balao et al., 2011); ^22^(Jürgens et al., 2002); ^23^(Dötterl et al., 2005); ^24^(Dötterl et al., 2012); ^25^(Zhuang et al., 2008); ^26^(Knudsen, 1991); ^27^(Bergström et al., 1991); ^28^(Knudsen and Klitgaard, 1998); ^29^(Lewis et al., 2003); ^30^(Pettersson and Knudsen, 2001); ^31^(Jiang et al., 2011); ^32^(Morinaga et al., 2008); ^33^(Azuma et al., 1997); ^34^(Azuma et al., 2001); ^35^(Levin et al., 2001); ^36^(Kite et al., 1991); ^37^(Raguso and Pichersky, 1995); ^38^(Barkman et al., 1997); ^39^(Bergström et al., 1992); ^40^(Johnson and Hobbhahn, 2010); ^41^(Huber et al., 2005); ^42^(Schiestl et al., 1997); ^43^(Awano et al., 1997); ^44^(Patt et al., 1988); ^45^(Olesen and Knudsen, 1994); ^46^(Okamoto et al., 2007); ^47^(Suchet et al., 2010); ^48^(Dötterl and Schäffler, 2007); ^49^(Steenhuisen et al., 2010); ^50^(Steenhuisen et al., 2012); ^51^(Rapparini et al., 2001); ^52^(Ômura et al., 1999); ^53^(Mactavish et al., 2000); ^54^(Parachnowitsch et al., 2012); ^55^(Loughrin et al., 1990); ^56^(Raguso et al., 2003); ^57^(Bernhardt et al., 2003); ^58^(Flamini et al., 2002).

| Acanthaceae | *Avicennia marina*^1^, *Razisea spicata*^2^ |
| --- | --- |
| Aceraceae | *Acer negundo*^3^ |
| Adoxaceae | *Sambucus* nigra^3^, *Viburnum lantana*^3^ |
| Aizoaceae | *Conophytum acutum*^4^, *C. angelicae*^4^, *C. armianum*^4^*, C. breve*^4^*, C. burgeri*^4^, *C. calculus*^4^, *C. carpianum*^4^, *C. concavum*^4^, *C. ficiforme*^4^, *C. fulleri*^4^, *C. hammeri*^4^, *C. hians*^4^, *C. jucundum*^4^, *C. lydiae*^4^, *C. maughanii*^4^, *C. obcordellum*^4^, *C. pageae*^4^, *C. piluliforme*^4^, *C. pubicalyx*^4^, *C. quaesitum*^4^, *C. regale*^4^, *C. saxetanum*^4^, *C. stephanii*^4^, *C. stevens-jonesianum*^4^, *C. subfenestratum*^4^, *C. truncatum*^4^, *C. uviforme*^4^ |
| Amaryllidaceae | *Crinum asiaticum* var. *japonicum*^5^, *Hippeastrum calyptratum*^6^, *Narcissus assoanus*^7^, *N. bugei*^7^, *N. bulbocodium*^7^, *N. cuatrecasasii*^7^, *N. gaditanus*^7^, *N. jonquilla*^7^, *N. papyraceus*^7^, *N. serotinus*^7^, *N. triandrus*^7^ |
| Anacardiaceae | *Pistacia lentiscus*^3^ |
| Annonaceae | *Anaxagorea brevipes*^8^, *A. dolichocarpa*^8^, *Duguetia asterotrichia*^8^, *Rollinia insignis*^8^, *Xylopia aromatica*^8^, *Xylopia benthamii*^8^ |
| Apocynaceae | *Cynanchum altiscandens*^9^, *C. auriculatum*^9^, *C. ellipticum*^9^, *C. formosum*^9^, *Funastrum cynanchoides*^9^, *F. elegans*^9^, *F. odoratum*^9^, *Gonolobus barbatus*^9^, *Metastelma cubense*^9^, *Orthosia scoparia*^9^, *Oxypetalum ostenii*^9^, *Plumeria alba*^10^, *Sarcostemma brevipedicellatum*^9^, *S. socotranum*^9^, *S. viminale*^9^, *Tabernaemontana amygdalifolia*^10^, *Vincetoxicum hirundinaria*^9^ |
| Araceae | *Anthurium armeniense*^11^, *A. fragantissimum*^11^, *A. lindenianum*^11^, *A. ochranthum*^11^, *A. roseospadix*^11^, *A. sagittatum*^12^, *A. thrinax*^12^, *Arum maculatum*^13^, *Caladium bicolor*^14^, *Philodendron acutatum*^14^, *Spathiphyllum humboldtii*^12^, *Taccarum ulei*^14^ |
| Arecaceae | *Calyptrogyne ghiesbreghtiana*^6^, *Geonoma macrostachys*^15^, *Nypa fruticans*^1^ |
| Asparagaceae | *Sansevieria cylindrica*^10^ |
| Asteraceae | *Calendula arvensis*^3^, *Centaurea scabiosa*^16^, *Chuquiraga jussieui*^2^, *Cirsium arvense*^16^, *Eupatorium cannabinum*^16^, *Helichrysum stoechas*^3^, *Inula salicina*^16^, *Mutisia alata*^2^, *Senecio articulatus*^17^ |
| Berberidaceae | *Berberis* sp.^2^, *Mahonia japonica*^18^ |
| Betulaceae | *Corylus avellana*^3^ |
| Bignoniaceae | *Crescentia cujete*^6^, *Parmentiera aculeata*^6^, *P. alata*^6^ |
| Brassicaceae | *Diplotaxis erucoides*^3^, *Lepidium draba*^3^ |
| Bromeliaceae | *Puya* sp.^2^, *Vriesea gladioliflora*^6^ |
| Cactaceae | *Echinopsis ancistrophora* ssp. *ancistrophora*^19^, *Weberocereus biolleyi*^6^, *W. tunilla*^6^ |
| Campanulaceae | *Siphocampylus giganteus*^2^ |
| Caprifoliaceae | *Centranthus ruber*^16^, *Knautia arvensis*^16^, *Lonicera japonica*^5^, *Scabiosa columbaria*^16^ |
| Caryophyllaceae | *Agrostemma githago*^16^, *Dianthus arenarius*^20^, *D. Armeria*^20^, *D. barbatus*^20^, *D. deltoides*^16,20^, *D. inoxianus*^21^, *D. monspessulanus*^20^, *D. superbus*^20^, *D. sylvestris*^20^, *Saponaria officinalis*^20^, *Silene chlorantha*^22^, *S. dichotoma*^22^, *S. flos-cuculi*^16^, *S. italica*^22^, *S. latifolia*^22,23^, *S. maritima*^10^, *S. nutans*^10,22^*, S. otites*^22,24^, *S. saxifraga*^22^, *S. sericea*^22^, *S. subconica*^22^, *S. succulenta*^22^, *S. vallesia*^22^, *S. viscosa*^22^, *S. vulgaris*^10,22^ |
| Combretaceae | *Lumnitzera racemosa*^1^ |
| Coriariaceae | *Coriaria myrtifolia*^3^ |
| Cornaceae | *Cornus florida*^25^ |
| Cucurbitaceae | *Trichosanthes kirilowii* var. *japonica*^5^ |
| Ericaceae | *Moneses uniflora*^26^, *Psammisia guianensis*^2^ |
| Euphorbiaceae | *Euphorbia characias*^3^ |
| Eupomatiaceae | *Eupomatia bennettii*^27^, *E. laurina*^27^ |
| Fabaceae | *Albizia julibrissin*^5^, *Bauhinia ungulata*^6^, *Brownea coccinea*^2^, *Browneopsis disepala*^28^, *Cyathostegia matthewsii*^29^, *Parkia biglobosa*^30^, *Wisteria floribunda*^31^, *W. sinensis*^31^ |
| Fagaceae | *Alnus glutinosa*^3^ |
| Gentianaceae | *Centaurium erythraea*^16^, *Macrocarpaea* sp.^2^, *Symbolanthus calygonus*^2^ |
| Gesneriaceae | *Gasteranthus oncogastrus*^2^ |
| Heliconiaceae | *Heliconia aemygdiana*^2^ |
| Lamiaceae | *Clerodendrum trichotomum*^5^, *Origanum vulgare*^16^, *Salvia verbenaca*^3^, *Thymus vulgaris*^3^ |
| Liliaceae | *Lilium auratum*^32^ |
| Loranthaceae | *Aetanthus andreanus*^2^, *Tristerix longebracteatus*^2^ |
| Lythraceae | *Pemphis acidula*^1^, *Sonneratia alba*^1^ |
| Magnoliaceae | *Liriodendron chinense*^33^, *L. tulipifera*^33^, *Magnolia acuminata*^33^, *M. figo*^33^, *M. grandiflora*^33^, *M. heptapeta*^33^, *M. hypoleuca*^33^, *M. kobus*^34^, *M. pyramidata*^33^, *M. salicifolia*^33^, *M. sieboldii* ssp. *japonica*^33^, *M. tamaulipana*^33^, *M. tripetala*^33^, *M. virginiana*^33^ |
| Malvaceae | *Tilia platyphyllos*^3^ |
| Nyctaginaceae | *Acleisanthes acutifolia*^35^, *A. crassifolia*^35^, *A. longiflora*^35^, *A. obtusa*^35^, *A. wrightii*^35^, *Mirabilis alipes*^35^, *M. bigelovii*^35^, *M. greenei*^35^, *M. jalapa*^35^, *M. longiflora*^35^, *M. macfarlanei*^35^, *M. multiflora*^35^, *M. pudica*^35^, *M. triflora*^35^, *Selinocarpus angustifolius*^35^, *S. chenopodioides*^35^, *S. lanceolatus*^35^, *S. parviflorus*^35^, *S. purpusianus*^35^, *S. undulatus*^35^ |
| Nymphaeaceae | *Victoria amazonica* x *cruziana*^36^ |
| Oleaceae | *Fraxinus angustifolia*^3^, *Ligustrum japonicum*^3^, *Olea europaea*^3^, *Syringa vulgaris*^3^ |
| Onagraceae | *Clarkia breweri*^37^, *C. concinna*^37^, *Fuchsia* sp.^2^, *Oenothera stricta*^5^ |
| Orchidaceae | *Anacamptis pyramidalis*^16^, *Cypripedium acaule*^38^, *C. arietinum*^38^, *C. calceolus*^39^, *C. candidum*^38^, *C. guttatum*^38^, *C. kentuckiense*^38^, *C. macranthum*^38^, *C. parviflorum*^38^, *C. pubescens*^38^, *C. reginae*^38^, *Disa fragrans*^40^, *D. sankeyi*^40^, *Epidendrum ciliare*^10^, *Gymnadenia conopsea*^41^, *G. odoratissima*^41^, *Ophrys sphegodes*^42^, *Phalaenopsis equestris*^43^, *P. schilleriana*^43^, *P. veithchiana*^43^, *Platanthera mandarinorum* var. *mandarinorum*^5^, *P. stricta*^44^ |
| Orobanchaceae | *Escobedia grandiflora*^10^ |
| Papaveraceae | *Corydalis cava*^45^ |
| Phyllanthaceae | *Glochidion acuminatum*^46^, *G. lanceolatum*^46^, *G. obovatum*^46^, *G. rubrum*^46^, *G. zeylanicum*^46^ |
| Plantaginaceae | *Antirrhinum majus ssp. pseudomajus*^47^, *A. majus* ssp *striatum*^47^ |
| Polemoniaceae | *Cobaea lutea*^2^*, C. scandens*^6^, *Phlox drummondii*^16^, *P. paniculata*^16^ |
| Primulaceae | *Lysimachia punctata*^48^, *Primula farinosa*^16^ |
| Proteaceae | *Oreocallis grandiflora*^2^*, Protea caffra*^49,50^, *P. cynaroides*^50^, *P. dracomontana*^49,50^, *P. laurifolia*^50^, *P. magnifica*^50^, *P. nitida*^50^, *P. punctata*^50^, *P. repens*^50^, *P. roupelliae*^50^, *P. simplex*^49,50^, *P. subvestita*^50^, *P. welwitschii*^49,50^ |
| Pyrolaceae | *Pyrola media*^26^, *P. norvegica*^26^, *P. rotundifolia*^26^ |
| Rhamnaceae | *Rhamnus alaternus*^3^ |
| Rhizophoraceae | *Bruguiera gymnorrhiza*^1^, *Kandelia candel*^1^, *Rhizophora stylosa*^1^ |
| Rosaceae | *Malus domestica*^51^, *Prunus avium*^51^, *P. dulcis*^3^, *P. yedoensis*^52^ |
| Rubiaceae | *Cephalanthus occidentalis*^16^, *Coussarea* sp. 1^10^, *Coussarea* sp. 2^10^, *Hillia parasitica*^10^, *Warszewiczia coccinea*^16^ |
| Rutaceae | *Boronia megastigma*^53^ |
| Scrophulariaceae | *Buddleja davidii*^16^, *Penstemon digitalis*^54^ |
| Solanaceae | *Brugmansia suaveolens*^10^, *Datura* sp.^10^, *Nicotiana alata*^55,56^, *Nicotiana bonariensis*^56^, *Nicotiana forgetiana*^56^, *Nicotiana langsdorffii*^56^, *Nicotiana longiflora*^56^, *Nicotiana plumbaginifolia*^56^, *Nicotiana rustica*^56^, *N. suaveolens*^55,56^, *N. sylvestris*^55,56^, *N. tomentosiformis*^55^, *Petunia axillaris*^43^ |
| Theophrastaceae | *Clavija euerganea*^12^, *C. repanda*^12^, *Deherainia smaragdina* ssp. *smaragdina*^12^, *Jacquinia keyensis*^12^, *J. macrocarpa*^12^, *J. sprucei*^12^ |
| Thymelaeaceae | *Daphne mezereum*^16^ |
| Trimeniaceae | *Trimenia moorei*^57^ |
| Ulmaceae | *Ulmus minor*^3^ |
| Verbenaceae | *Lantana camara*^16^ |
| Violaceae | *Viola etrusca*^58^ |
| Zingiberaceae | *Hedychium coronarium*^10^ |

**Reference list**

Andersson, S., Nilsson, L.A.A., Groth, I., Bergstrom, G., 2002. Floral scents in butterfly-pollinated plants: possible convergence in chemical composition. Bot. J. Linn. Soc. 140, 129–153. doi:10.1046/j.1095-8339.2002.00068.x

Awano, K., Honda, T., Ogawa, T., Suzuki, S., Matsunaga, Y., 1997. Volatile Components of *Phalaenopsis schilleriana* Rehb. f. Flavour Fragr. J. 12, 341–344.

Azuma, H., Toyota, M., Asakawa, Y., 2001. lntraspecific Variation of Floral Scent Chemistry in *Magnolia kobus* DC. (Magnoliaceae). J. Plant Res. 114, 411–422.

Azuma, H., Toyota, M., Asakawa, Y., Takaso, T., Tobe, H., 2002. Floral scent chemistry of mangrove plants. J. Plant Res. 115, 47–53.

Azuma, H., Toyota, M., Asakawa, Y., Yamaoka, R., Garcia-Franco, J.G., Dieringer, G., Thien, L.B., Kawano, S., 1997. Chemical divergence in Floral Scents of *Magnolia* and Allied Genera (Magnoliaceae). Plant Species Biol. 12, 69–83.

Balao, F., Herrera, J., Talavera, S., Dötterl, S., 2011. Spatial and temporal patterns of floral scent emission in *Dianthus inoxianus* and electroantennographic responses of its hawkmoth pollinator. Phytochemistry 72, 601–609. doi:10.1016/j.phytochem.2011.02.001

Barkman, T.J., Beaman, J.H., Gaget, D.A., 1997. Floral fragrance variation in *Cypripedium*: implications for evolutionary and ecological studies. Phytochemistry 44, 875–882.

Bergström, G., Birgersson, G., Groth, I., Nilsson, A., 1992. Floral fragrance disparity between three taxa of lady’s slipper *Cypripedium calceolus* (Orchidaceae). Phytochemistry 31, 2315–2319.

Bergström, G., Dobson, H.E.M., Groth, I., Pellmyr, O., Endress, P.K., Thien, L.B., Hiibener, A., Francke, W., 1991. Chemical basis of a highly specific mutualism: chiral esters attract pollinating beetles in Eupomatiaceae. Phytochemistry 30, 3221–3225.

Bernhardt, P., Sage, T., Weston, P., Azuma, H., Lam, M., Thien, L.B., Bruhl, J., 2003. The pollination of *Trimenia moorei* (Trimeniaceae): floral volatiles, insect/wind pollen vectors and stigmatic self-incompatibility in a basal angiosperm. Ann. Bot. 92, 445–458. doi:10.1093/aob/mcg157

Bestmann, H.C.E.N., 1997. Headspace analysis of volatile flower scent conastituents of bat-pollinated plants. Phytochemstry 46, 1169–1172.

Dobson, H.E.M., Arroyo, T.J., Mt, G.B., Grotht, I., 1997. Interspecific Variation in Floral Fragrances within the Genus *Narcissus* (Amaryllidaceae). Biochem. Syst. Ecol. 25, 685–706.

Dötterl, S., Jahreiß, K., Jhumur, U.S., Jürgens, A., 2012. Temporal variation of flower scent in *Silene otites* (Caryophyllaceae): a species with a mixed pollination system. Bot. J. Linn. Soc. 169, 447–460.

Dötterl, S., Schäffler, I., 2007. Flower scent of floral oil-producing *Lysimachia punctata* as attractant for the oil-bee *Macropis fulvipes*. J. Chem. Ecol. 33, 441–445. doi:10.1007/s10886-006-9237-2

Dötterl, S., Wolfe, L.M., Jürgens, A., 2005. Qualitative and quantitative analyses of flower scent in *Silene latifolia*. Phytochemistry 66, 203–213. doi:10.1016/j.phytochem.2004.12.002

Farré-Armengol, G., Filella, I., Llusià, J., Peñuelas, J., 2015. Pollination mode determines floral scent. Biochem. Syst. Ecol. 61, 44–53. doi:10.1016/j.bse.2015.05.007

Flamini, G., Cioni, P.L., Morelli, I., 2002. Analysis of the essential oil of the aerial parts of *Viola etrusca* from Monte Labbro (South Tuscany, Italy) andin vivo analysis of flower volatiles using SPME. Flavour Fragr. J. 17, 147–149. doi:10.1002/ffj.1060

Hentrich, H., Kaiser, R., Gottsberger, G., 2010. Floral biology and reproductive isolation by floral scent in three sympatric aroid species in French Guiana. Plant Biol. 12, 587–596. doi:10.1111/j.1438-8677.2009.00256.x

Huber, F.K., Kaiser, R., Sauter, W., Schiestl, F.P., 2005. Floral scent emission and pollinator attraction in two species of *Gymnadenia* (Orchidaceae). Oecologia 142, 564–75. doi:10.1007/s00442-004-1750-9

Jiang, Y., Chen, X., Lin, H., Wang, F., Chen, F., 2011. Floral Scent in *Wisteria*: Chemical Composition, Emission Pattern, and Regulation. J. Am. Soc. Hortic. Sci. 136, 307–314.

Johnson, S.D., Hobbhahn, N., 2010. Generalized pollination, floral scent chemistry, and a possible case of hybridization in the African orchid *Disa fragrans*. South African J. Bot. 76, 739–748. doi:10.1016/j.sajb.2010.07.008

Jürgens, A., 2004. Nectar sugar composition and floral scent compounds of diurnal and nocturnal *Conophytum species* (Aizoaceae). South African J. Bot. 70, 191–205. doi:10.1016/S0254-6299(15)30235-0

Jürgens, A., Dötterl, S., Liede-Schumann, S., Meve, U., 2009. Chemical diversity of floral volatiles in Asclepiadoideae-Asclepiadeae (Apocynaceae). Biochem. Syst. Ecol. 36, 842–852. doi:10.1016/j.bse.2008.10.005

Jürgens, A., Webber, A.C., Gottsberger, G., 2000. Floral scent compounds of Amazonian Annonaceae species pollinated by small beetles and thrips. Phytochemistry 55, 551–558.

Jürgens, A., Witt, T., Gottsberger, G., 2003. Flower scent composition in *Dianthus* and *Saponaria* species (Caryophyllaceae) and its relevance for pollination biology and taxonomy. Biochem. Syst. Ecol. 31, 345–357. doi:10.1016/S0305-1978(02)00173-4

Jürgens, A., Witt, T., Gottsberger, G., 2002. Flower scent composition in night-flowering *Silene* species (Caryophyllaceae). Biochem. Syst. Ecol. 30, 383–397.

Kite, G., Reynolds, T., Prance, G.T., 1991. Potential Pollinator-attracting Chemicals from *Victoria* (Nymphaeaceae). Biochem. Syst. Ecol. 19, 535–539.

Kite, G.C., 1995. The Floral Odour of *Arum maculatum*. Biochem. Syst. Ecol. 23, 343–354.

Kite, G.C., Smith, S.A.L., 1997. Inflorescence odour of *Senecio articulatus*: temporal variation in isovaleric acid levels. Phtyochemistry 45, 1135–1138.

Knudsen, J.T., 2002. Variation in floral scent composition within and between populations of *Geonoma macrostachys* (Arecaceae) in the western Amazon. Am. J. Bot. 89, 1772–1778.

Knudsen, J.T., 1991. Floral scent and intrafloral scent differentiation in *Moneses* and *Pyrola* (Pyrolaceae). Plant Syst. Evol. 177, 81–91. doi:10.1111/j.1756-1051.1994.tb00599.x

Knudsen, J.T., Klitgaard, B.B., 1998. Floral scent and pollination in *Browneopsis disepala* (Leguminosae: Caesalpinioideae) in western Ecuador. Brittonia 50, 174–182.

Knudsen, J.T., Tollsten, L., 1993. Trends in floral scent chemistry in pollination syndromes: floral scent composition in moth-pollinated taxa. Bot. J. Linn. Soc. 113, 263–284. doi:10.1111/j.1095-8339.1993.tb00340.x

Knudsen, J.T., Tollsten, L., Groth, I., Bergström, G., Raguso, R.A., 2004. Trends in floral scent chemistry in pollination syndromes: floral scent composition in hummingbird-pollinated taxa. Bot. J. Linn. Soc. 146, 191–199.

Kuanprasert, N., Kuehnle, A.R., Tang, C.S., 1998. Floral fragrance compounds of some *Anthurium* (Araceae) species and hybrids. Phytochemistry 49, 521–528.

Levin, R.A., Raguso, R.A., McDade, L.A., 2001. Fragrance chemistry and pollinator affinities in Nyctaginaceae. Phytochemistry 58, 429–440.

Lewis, G.P., Knudsen, J.T., Klitgaard, B.B., Pennington, R.T., 2003. The floral scent of *Cyathostegia mathewsii* (Leguminosae, Papilionoideae) and preliminary observations on reproductive biology. Biochem. Syst. Ecol. 31, 951–962. doi:10.1016/S0305-1978(03)00045-0

Loughrin, J.H., Hamilton-Kemp, T.R., Andersen, R.A., Hildebrand, D.F., 1990. Headspace Compounds from Flowers of *Nicotiana tabacum* and Related Species. J. Agric. Food Chem. 38, 455–460.

Mactavish, H.S., Davies, N.W., Menary, R.C., 2000. Emission of Volatiles From Brown *Boronia* Flowers: Some Comparative Observations. Ann. Bot. 86, 347–354. doi:10.1006/anbo.2000.1194

Maia, A.C.D., Gibernau, M., Dötterl, S., Navarro, D.M.D.A.F., Seifert, K., Müller, T., Schlindwein, C., 2013. The floral scent of *Taccarum ulei* (Araceae): attraction of scarab beetle pollinators to an unusual aliphatic acyloin. Phytochemistry 93, 71–78. doi:10.1016/j.phytochem.2013.03.005

Miyake, T., Yamaoka, R., Yahara, T., 1998. Floral Scents of Hawkmoth-Pollinated Flowers in Japan. J. Plant Res. 111, 199–205.

Morinaga, S., Kumano, Y., Ota, A., Yamaoka, R., Sakai, S., 2008. Day-night fluctuations in floral scent and their effects on reproductive success in *Lilium auratum*. Popul. Ecol. 51, 187–195.

Okamoto, T., Kawakita, A., Kato, M., 2007. Interspecific variation of floral scent composition in *Glochidion* and its association with host-specific pollinating seed parasite (*Epicephala*). J. Chem. Ecol. 33, 1065–1081. doi:10.1007/s10886-007-9287-0

Olesen, J.M., Knudsen, J.T., 1994. Scent Profiles of Flower Colour Morphs of *Corydalis cava* (Fumariaceae) in Relation to Foraging Behaviour of Bumblebee Queens (*Bombus terrestris*) 22, 231–237.

Ômura, H., Honda, K., Nakagawa, A., Hayashi, N., 1999. The role of floral scent of the cherry tree, *Prunus yedoensis*, in the foraging behavior of *Luehdorfia japonica* (Lepidoptera: Papilionidae). Appl. Entomol. Zool. 34, 309–313.

Parachnowitsch, A.L., Raguso, R.A., Kessler, A., 2012. Phenotypic selection to increase floral scent emission, but not flower size or colour in bee-pollinated *Penstemon digitalis*. New Phytol. 195, 667–675. doi:10.1111/j.1469-8137.2012.04188.x

Patt, J.M., Rhoades, D.F., Corkill, J.A., 1988. Analysis of the floral fragrance of *Platanthera stricta*. Phytochemistry 27, 91–95.

Pettersson, S., Knudsen, J.T., 2001. Floral scent and nectar production in *Parkia biglobosa* Jacq. (Leguminosae: Mimosoideae). Bot. J. Linn. Soc. 135, 97–106. doi:10.1006/boj1.2000.0402

Picone, J.M., Mactavish, H.S., Clery, R.A., 2002. Emission of floral volatiles from *Mahonia japonica* (Berberidaceae). Phytochemistry 60, 611–617.

Raguso, R.A., Levin, R.A., Foose, S.E., Holmberg, M.W., McDade, L.A., 2003. Fragrance chemistry, nocturnal rhythms and pollination “syndromes” in *Nicotiana*. Phytochemistry 63, 265–284.

Raguso, R.A., Pichersky, E., 1995. Floral volatiles from *Clarkia breweri* and *C. concinna* (Onagraceae): recent evolution of floral scent and moth pollination. Plant Syst. Evol. 194, 55–67.

Rapparini, F., Baraldi, R., Facini, O., 2001. Seasonal variation of monoterpene emission from *Malus domestica* and *Prunus avium*. Phytochemistry 57, 681–687.

Schiestl, F.P., Ayasse, M., Paulus, H.F., Erdmann, D., Francke, W., 1997. Variation of floral scent emission and postpollination changes in individual flowers of *Ophrys sphegodes* subsp. *sphegodes*. J. Chem. Ecol. 23, 2881–2895.

Schlumpberger, B.O., Raguso, R.A., 2008. Geographic variation in floral scent of *Echinopsis ancistrophora* (Cactaceae); evidence for constraints on hawkmoth attraction. Oikos 117, 801–814. doi:10.1111/j.2008.0030-1299.16211.x

Steenhuisen, S.-L., Raguso, R.A., Jürgens, A., Johnson, S.D., 2010. Variation in scent emission among floral parts and inflorescence developmental stages in beetle-pollinated *Protea* species (Proteaceae). South African J. Bot. 76, 779–787. doi:10.1016/j.sajb.2010.08.008

Steenhuisen, S.-L., Raguso, R. a, Johnson, S.D., 2012. Floral scent in bird- and beetle-pollinated *Protea* species (Proteaceae): chemistry, emission rates and function. Phytochemistry 84, 78–87. doi:10.1016/j.phytochem.2012.08.012

Suchet, C., Dormont, L., Schatz, B., Giurfa, M., Simon, V., Raynaud, C., Chave, J., 2010. Floral scent variation in two *Antirrhinum majus* subspecies influences the choice of naïve bumblebees. Behav. Ecol. Sociobiol. 65, 1015–1027. doi:10.1007/s00265-010-1106-x

Zhuang, X., Klingeman, W.E., Hu, J., Chen, F., 2008. Emission of Volatile Chemicals from Flowering Dogwood (*Cornus* *florida* L.) Flowers. J. Agric. Food Chem. 56, 9570–9574.
