# Supplementary material for: Deciphering the Biotic and Climatic Factors That Influence Floral Scents: A Systematic Review of Floral Volatile Emissions
Source: Front Plant Sci. 2020 Jul 31;11:1154. doi: 10.3389/fpls.2020.01154 (PMC7412988; doi:10.3389/fpls.2020.01154)
Supplement: Supplementary file 8 [file Table_2.docx]

**Table S2.** Complete list of all floral volatiles that were identified in the floral scents of plant species included in this work (N = 305, species are listed in **Table S1**) classified by chemical groups. The table provides the percentage of species in which each compound was present and the percentage of species where the compound represented >25, >50, and >75% of the total floral emissions. The percentage of species where the chemical group was present and the percentage of species with emission percentages >25, >50, and >75% of the total floral emissions are provided.

|  | **Presence**  **(%)** | **Species with >25% (%)** | **Species with >50% (%)** | **Species with >75% (%)** |
| --- | --- | --- | --- | --- |
| **FATTY ACID DERIVATIVES** | **77.38** | **23.28** | **12.46** | **5.90** |
| (Z)-3-hexen-1-ol | 22.62 | 0.33 | 0.33 | 0.00 |
| (Z)-3-hexenyl acetate | 20.00 | 1.97 | 0.33 | 0.00 |
| hexadecane | 9.51 | 0.00 | 0.00 | 0.00 |
| 2-decen-1-ol | 8.85 | 0.00 | 0.00 | 0.00 |
| decanal | 8.52 | 0.33 | 0.00 | 0.00 |
| 3-nonen-1-ol | 8.52 | 0.00 | 0.00 | 0.00 |
| nonanal | 8.20 | 0.33 | 0.00 | 0.00 |
| octanal | 7.54 | 0.00 | 0.00 | 0.00 |
| 1-hexanol | 6.89 | 0.00 | 0.00 | 0.00 |
| n-decanal | 5.90 | 0.00 | 0.00 | 0.00 |
| pentadecane | 5.57 | 0.66 | 0.66 | 0.33 |
| heptanal | 5.57 | 0.00 | 0.00 | 0.00 |
| n-octanal | 5.57 | 0.00 | 0.00 | 0.00 |
| n-nonanal | 5.25 | 0.00 | 0.00 | 0.00 |
| tetradecane | 4.92 | 0.66 | 0.33 | 0.00 |
| 1-octen-3-ol | 4.92 | 0.00 | 0.00 | 0.00 |
| methyl hexadecanoate | 4.92 | 0.00 | 0.00 | 0.00 |
| (Z)-3-hexenyl 2-methyl butanoate | 4.26 | 0.00 | 0.00 | 0.00 |
| nonanoic acid | 4.26 | 0.00 | 0.00 | 0.00 |
| 3-hexenyl ester butanoic acid | 3.93 | 0.00 | 0.00 | 0.00 |
| 3-hydroxy-2-butanone (acetoin) | 3.93 | 0.00 | 0.00 | 0.00 |
| 7-octen-4-ol | 3.61 | 0.00 | 0.00 | 0.00 |
| dodecane | 3.61 | 0.00 | 0.00 | 0.00 |
| decanoic acid | 3.28 | 0.00 | 0.00 | 0.00 |
| hexanal | 3.28 | 0.00 | 0.00 | 0.00 |
| hexyl acetate | 3.28 | 0.00 | 0.00 | 0.00 |
| n-heptanal | 3.28 | 0.00 | 0.00 | 0.00 |
| tridecane | 3.28 | 0.00 | 0.00 | 0.00 |
| (E)-2-hexenal | 2.95 | 0.33 | 0.33 | 0.00 |
| heptadecane | 2.95 | 0.00 | 0.00 | 0.00 |
| hexanol | 2.95 | 0.00 | 0.00 | 0.00 |
| n-hexadecanoic acid | 2.62 | 1.31 | 0.00 | 0.00 |
| 2-heptanone | 2.62 | 0.33 | 0.00 | 0.00 |
| (Z)-3-hexenyl tiglate | 2.62 | 0.33 | 0.00 | 0.00 |
| 3-methyl-2-pentanone | 2.62 | 0.00 | 0.00 | 0.00 |
| (E)-2-hexen-1-ol | 2.62 | 0.00 | 0.00 | 0.00 |
| n-octanol | 2.62 | 0.00 | 0.00 | 0.00 |
| octadecanoic acid | 2.62 | 0.00 | 0.00 | 0.00 |
| (E)-2-hexenyl acetate | 2.30 | 0.00 | 0.00 | 0.00 |
| acetic acid | 2.30 | 0.00 | 0.00 | 0.00 |
| hexanoic acid | 2.30 | 0.00 | 0.00 | 0.00 |
| methyl decanoate | 2.30 | 0.00 | 0.00 | 0.00 |
| pentadecanoic acid | 2.30 | 0.00 | 0.00 | 0.00 |
| undecane | 2.30 | 0.00 | 0.00 | 0.00 |
| 2-heptanol | 1.97 | 0.33 | 0.00 | 0.00 |
| 2-nonenal | 1.97 | 0.00 | 0.00 | 0.00 |
| methyl hexanoate | 1.97 | 0.00 | 0.00 | 0.00 |
| methyl octanoate | 1.97 | 0.00 | 0.00 | 0.00 |
| tetradecanoic acid | 1.97 | 0.00 | 0.00 | 0.00 |
| methyl 2-methylbutanoate | 1.64 | 0.33 | 0.33 | 0.00 |
| 1-octanol | 1.64 | 0.00 | 0.00 | 0.00 |
| 2-nonanone | 1.64 | 0.00 | 0.00 | 0.00 |
| (Z)-3-hexen-1-ol acetate | 1.64 | 0.00 | 0.00 | 0.00 |
| (Z)-3-hexenyl 3-methylbutanoate | 1.64 | 0.00 | 0.00 | 0.00 |
| decane | 1.64 | 0.00 | 0.00 | 0.00 |
| E-9-octadecenoic acid | 1.64 | 0.00 | 0.00 | 0.00 |
| heptanoic acid | 1.64 | 0.00 | 0.00 | 0.00 |
| n-hexanal | 1.64 | 0.00 | 0.00 | 0.00 |
| n-hexyl acetate | 1.64 | 0.00 | 0.00 | 0.00 |
| octanol | 1.64 | 0.00 | 0.00 | 0.00 |
| γ-butyrolactone | 1.64 | 0.00 | 0.00 | 0.00 |
| 3-hexanone | 1.31 | 0.66 | 0.00 | 0.00 |
| butyl acetate | 1.31 | 0.33 | 0.33 | 0.33 |
| 2-tridecanone | 1.31 | 0.00 | 0.00 | 0.00 |
| 3-hexenyl ester hexanoic acid | 1.31 | 0.00 | 0.00 | 0.00 |
| 7-decen-5-olide (jasmin lactone) | 1.31 | 0.00 | 0.00 | 0.00 |
| (E)-3-hexen-1-ol | 1.31 | 0.00 | 0.00 | 0.00 |
| dodecanoic acid | 1.31 | 0.00 | 0.00 | 0.00 |
| dodecanol | 1.31 | 0.00 | 0.00 | 0.00 |
| ethyl hexanoate | 1.31 | 0.00 | 0.00 | 0.00 |
| methyl decenoate | 1.31 | 0.00 | 0.00 | 0.00 |
| δ-octalactone | 1.31 | 0.00 | 0.00 | 0.00 |
| dodecyl acetate | 0.98 | 0.33 | 0.00 | 0.00 |
| heptadecene | 0.98 | 0.33 | 0.00 | 0.00 |
| 2-nonanol | 0.98 | 0.00 | 0.00 | 0.00 |
| 2-undecanone | 0.98 | 0.00 | 0.00 | 0.00 |
| 2,3-butanedione | 0.98 | 0.00 | 0.00 | 0.00 |
| 3-hexen-2-one | 0.98 | 0.00 | 0.00 | 0.00 |
| 3-hexenyl ester heptanoic acid | 0.98 | 0.00 | 0.00 | 0.00 |
| (Z)-3-hexenyl butyrate | 0.98 | 0.00 | 0.00 | 0.00 |
| (Z)-3-hexenyl propionate | 0.98 | 0.00 | 0.00 | 0.00 |
| (Z)-4-hexenyl acetate | 0.98 | 0.00 | 0.00 | 0.00 |
| dodecanal | 0.98 | 0.00 | 0.00 | 0.00 |
| hexyl butanoate | 0.98 | 0.00 | 0.00 | 0.00 |
| hexyl pentanoate | 0.98 | 0.00 | 0.00 | 0.00 |
| methyl 5-methylheptanoate | 0.98 | 0.00 | 0.00 | 0.00 |
| methyl butanoate | 0.98 | 0.00 | 0.00 | 0.00 |
| methyl hexenoate | 0.98 | 0.00 | 0.00 | 0.00 |
| methyl pentanoate | 0.98 | 0.00 | 0.00 | 0.00 |
| nonane | 0.98 | 0.00 | 0.00 | 0.00 |
| nonanol | 0.98 | 0.00 | 0.00 | 0.00 |
| octadecane | 0.98 | 0.00 | 0.00 | 0.00 |
| pentadecene | 0.98 | 0.00 | 0.00 | 0.00 |
| tetradecene | 0.98 | 0.00 | 0.00 | 0.00 |
| γ-caprolactone | 0.98 | 0.00 | 0.00 | 0.00 |
| 2-hydroxy-5-methyl-3-hexanone | 0.66 | 0.33 | 0.33 | 0.33 |
| ethyl acetate | 0.66 | 0.33 | 0.33 | 0.00 |
| 1,3(E),5(Z)-undecatriene | 0.66 | 0.33 | 0.00 | 0.00 |
| (Z)-8-heptadecene | 0.66 | 0.33 | 0.00 | 0.00 |
| decanoic acid methyl ester | 0.66 | 0.33 | 0.00 | 0.00 |
| decyl acetate | 0.66 | 0.33 | 0.00 | 0.00 |
| ethyl 3-methylbutanoate | 0.66 | 0.33 | 0.00 | 0.00 |
| methyl tiglate | 0.66 | 0.33 | 0.00 | 0.00 |
| octanoic acid methyl ester | 0.66 | 0.33 | 0.00 | 0.00 |
| 1-octen-3-ol acetate | 0.66 | 0.00 | 0.00 | 0.00 |
| 1,3(E),5(E)-undecatriene | 0.66 | 0.00 | 0.00 | 0.00 |
| 2-ethylhexanol | 0.66 | 0.00 | 0.00 | 0.00 |
| 2-hydroxyhexan-2-one | 0.66 | 0.00 | 0.00 | 0.00 |
| 2-pentadecanone | 0.66 | 0.00 | 0.00 | 0.00 |
| 2-phenylethyl 3-methylbutanoate | 0.66 | 0.00 | 0.00 | 0.00 |
| 2,3-butanediol | 0.66 | 0.00 | 0.00 | 0.00 |
| 2,6-nonadienal | 0.66 | 0.00 | 0.00 | 0.00 |
| 3-hydroxy-5-methyl-2-hexanone | 0.66 | 0.00 | 0.00 | 0.00 |
| 4-decenoic acid methyl ester | 0.66 | 0.00 | 0.00 | 0.00 |
| 4-methylhexanoic acid | 0.66 | 0.00 | 0.00 | 0.00 |
| 5,9,13-trimethyl-4,8,12-tetradecatrienal | 0.66 | 0.00 | 0.00 | 0.00 |
| (E)-2-octenal | 0.66 | 0.00 | 0.00 | 0.00 |
| (Z)-3-hexenal | 0.66 | 0.00 | 0.00 | 0.00 |
| cyclohexanone | 0.66 | 0.00 | 0.00 | 0.00 |
| dimethylcyclohexanone isomer | 0.66 | 0.00 | 0.00 | 0.00 |
| ethyl butanoate | 0.66 | 0.00 | 0.00 | 0.00 |
| ethyl tiglate | 0.66 | 0.00 | 0.00 | 0.00 |
| heptadecadiene | 0.66 | 0.00 | 0.00 | 0.00 |
| heptan-1-ol | 0.66 | 0.00 | 0.00 | 0.00 |
| hexadecene | 0.66 | 0.00 | 0.00 | 0.00 |
| hexyl butyrate | 0.66 | 0.00 | 0.00 | 0.00 |
| isoamyl isovalerate | 0.66 | 0.00 | 0.00 | 0.00 |
| isobutyric acid | 0.66 | 0.00 | 0.00 | 0.00 |
| isopropyl myristate (isopropyl tetradecanoate) | 0.66 | 0.00 | 0.00 | 0.00 |
| methyl-(2S)-2-hydroxy-3-methylbutanoate | 0.66 | 0.00 | 0.00 | 0.00 |
| methyl-(2R,3S)-2-hydroxy-3-methylpentanoate | 0.66 | 0.00 | 0.00 | 0.00 |
| methyl-(2S,3S)-2-hydroxy-3-methylpentanoate | 0.66 | 0.00 | 0.00 | 0.00 |
| methyl 2-hexenoate | 0.66 | 0.00 | 0.00 | 0.00 |
| methyl 4-methylhexanoate | 0.66 | 0.00 | 0.00 | 0.00 |
| methyl 5-methylhexanoate | 0.66 | 0.00 | 0.00 | 0.00 |
| methyl dodecanoate | 0.66 | 0.00 | 0.00 | 0.00 |
| methyl isobutenyl ketone | 0.66 | 0.00 | 0.00 | 0.00 |
| n-decanoic acid | 0.66 | 0.00 | 0.00 | 0.00 |
| n-pentadecane | 0.66 | 0.00 | 0.00 | 0.00 |
| n-tetradecane | 0.66 | 0.00 | 0.00 | 0.00 |
| n-undecane | 0.66 | 0.00 | 0.00 | 0.00 |
| nonadecane | 0.66 | 0.00 | 0.00 | 0.00 |
| nonan-2-one | 0.66 | 0.00 | 0.00 | 0.00 |
| nonanoic acid methyl ester | 0.66 | 0.00 | 0.00 | 0.00 |
| octanoic acid | 0.66 | 0.00 | 0.00 | 0.00 |
| pentyl acetate | 0.66 | 0.00 | 0.00 | 0.00 |
| propionic acid | 0.66 | 0.00 | 0.00 | 0.00 |
| tetradecanal | 0.66 | 0.00 | 0.00 | 0.00 |
| tetradecyl acetate | 0.66 | 0.00 | 0.00 | 0.00 |
| γ-vinyl-γ-valerolactone | 0.66 | 0.00 | 0.00 | 0.00 |
| δ-nonalactone | 0.66 | 0.00 | 0.00 | 0.00 |
| methyl-(2S)-2-methylbutanoate | 0.33 | 0.33 | 0.33 | 0.33 |
| ethyl 2-methylbutanoate | 0.33 | 0.33 | 0.33 | 0.00 |
| methyl-(2R)-2-acetoxy-4-methylpentanoate | 0.33 | 0.33 | 0.33 | 0.00 |
| 1-hexadecyl acetate | 0.33 | 0.33 | 0.00 | 0.00 |
| 2-undecyl acetate | 0.33 | 0.33 | 0.00 | 0.00 |
| isovaleric acid | 0.33 | 0.33 | 0.00 | 0.00 |
| octyl acetate | 0.33 | 0.33 | 0.00 | 0.00 |
| 1-butyl acetate | 0.33 | 0.00 | 0.00 | 0.00 |
| 1-hexyl acetate | 0.33 | 0.00 | 0.00 | 0.00 |
| 1-methylbutyl acetate | 0.33 | 0.00 | 0.00 | 0.00 |
| 1-octene | 0.33 | 0.00 | 0.00 | 0.00 |
| 1-pentanol | 0.33 | 0.00 | 0.00 | 0.00 |
| 1,1-diethoxyethane | 0.33 | 0.00 | 0.00 | 0.00 |
| 1,14-tetradecanediol | 0.33 | 0.00 | 0.00 | 0.00 |
| 1,3,5-cycloheptatriene | 0.33 | 0.00 | 0.00 | 0.00 |
| 2-butanone | 0.33 | 0.00 | 0.00 | 0.00 |
| 2-heptyl acetate | 0.33 | 0.00 | 0.00 | 0.00 |
| 2-hexenol | 0.33 | 0.00 | 0.00 | 0.00 |
| 2-hexenol acetate | 0.33 | 0.00 | 0.00 | 0.00 |
| 2-hexenyl ester butanoic acid | 0.33 | 0.00 | 0.00 | 0.00 |
| 2-hydroxyheptan-3-one | 0.33 | 0.00 | 0.00 | 0.00 |
| 2-octanol | 0.33 | 0.00 | 0.00 | 0.00 |
| 2-octanone | 0.33 | 0.00 | 0.00 | 0.00 |
| 2-tridecyl acetate | 0.33 | 0.00 | 0.00 | 0.00 |
| 2,3-heptanedione | 0.33 | 0.00 | 0.00 | 0.00 |
| 3-decenoic acid ethyl ester | 0.33 | 0.00 | 0.00 | 0.00 |
| 3-hydroxyheptan-2-one | 0.33 | 0.00 | 0.00 | 0.00 |
| 3-methyl-2-hexanone | 0.33 | 0.00 | 0.00 | 0.00 |
| 3-methylpentanol | 0.33 | 0.00 | 0.00 | 0.00 |
| 3-octenoic acid ehtyl ester | 0.33 | 0.00 | 0.00 | 0.00 |
| 3,6-dodecadienoic acid methyl ester | 0.33 | 0.00 | 0.00 | 0.00 |
| 3,6-heptanedione | 0.33 | 0.00 | 0.00 | 0.00 |
| 4-methylhexanol | 0.33 | 0.00 | 0.00 | 0.00 |
| 4-methylpentanol | 0.33 | 0.00 | 0.00 | 0.00 |
| 4-octenoic acid methyl ester | 0.33 | 0.00 | 0.00 | 0.00 |
| 5-hepten-2-one | 0.33 | 0.00 | 0.00 | 0.00 |
| 5-methylhexane-2,3-dione | 0.33 | 0.00 | 0.00 | 0.00 |
| 6-methyl-5-hepten-2-one | 0.33 | 0.00 | 0.00 | 0.00 |
| 6-methylheptanol | 0.33 | 0.00 | 0.00 | 0.00 |
| (E)-2-dodecenal | 0.33 | 0.00 | 0.00 | 0.00 |
| (E)-nonen-2-al | 0.33 | 0.00 | 0.00 | 0.00 |
| (E)-3-hexenyl butyrate | 0.33 | 0.00 | 0.00 | 0.00 |
| (E)-7-methyl-1,6-dioxaspiro-[4,5]decane | 0.33 | 0.00 | 0.00 | 0.00 |
| (E,E)-2,4-hexadienal | 0.33 | 0.00 | 0.00 | 0.00 |
| (Z)-3-hexenyl ethanoate | 0.33 | 0.00 | 0.00 | 0.00 |
| (Z)-3-hexenyl isovalerate | 0.33 | 0.00 | 0.00 | 0.00 |
| (Z)-3-hexenyl propanoate | 0.33 | 0.00 | 0.00 | 0.00 |
| (Z)-6-nonen-1-ol | 0.33 | 0.00 | 0.00 | 0.00 |
| (Z)-6-nonenal | 0.33 | 0.00 | 0.00 | 0.00 |
| (Z)-9-hecadecenal | 0.33 | 0.00 | 0.00 | 0.00 |
| (Z)-n-heptadec-8-ene | 0.33 | 0.00 | 0.00 | 0.00 |
| azulene | 0.33 | 0.00 | 0.00 | 0.00 |
| butyl butanoate | 0.33 | 0.00 | 0.00 | 0.00 |
| decanoic acid ethyl ester | 0.33 | 0.00 | 0.00 | 0.00 |
| decanol | 0.33 | 0.00 | 0.00 | 0.00 |
| dodecanoic acid ethyl ester | 0.33 | 0.00 | 0.00 | 0.00 |
| dodecanoic acid methyl ester | 0.33 | 0.00 | 0.00 | 0.00 |
| eicosane | 0.33 | 0.00 | 0.00 | 0.00 |
| ethyl 3-hexanoate | 0.33 | 0.00 | 0.00 | 0.00 |
| ethyl hexadecanoate | 0.33 | 0.00 | 0.00 | 0.00 |
| heptyl acetate | 0.33 | 0.00 | 0.00 | 0.00 |
| hexadecanoic acid | 0.33 | 0.00 | 0.00 | 0.00 |
| hexadecyl acetate | 0.33 | 0.00 | 0.00 | 0.00 |
| hexenyl tiglate | 0.33 | 0.00 | 0.00 | 0.00 |
| iso-pentyl butanoate | 0.33 | 0.00 | 0.00 | 0.00 |
| isoamyl n-propanoate | 0.33 | 0.00 | 0.00 | 0.00 |
| isoamyl tiglate | 0.33 | 0.00 | 0.00 | 0.00 |
| isobutyl (Z)-2-methyl-2-butenoate | 0.33 | 0.00 | 0.00 | 0.00 |
| isobutyl 2-methylbutanoate | 0.33 | 0.00 | 0.00 | 0.00 |
| isobutyl acetate | 0.33 | 0.00 | 0.00 | 0.00 |
| isobutyl hexanoate | 0.33 | 0.00 | 0.00 | 0.00 |
| isobutyl isobutanoate | 0.33 | 0.00 | 0.00 | 0.00 |
| isobutyl isovalerate | 0.33 | 0.00 | 0.00 | 0.00 |
| isobutyl tiglate | 0.33 | 0.00 | 0.00 | 0.00 |
| isopentyl 3-methylbutanoate | 0.33 | 0.00 | 0.00 | 0.00 |
| isopropyl propanoate | 0.33 | 0.00 | 0.00 | 0.00 |
| isopropyl tiglate | 0.33 | 0.00 | 0.00 | 0.00 |
| methacrolein | 0.33 | 0.00 | 0.00 | 0.00 |
| methyl-(2R)-2-acetoxy-3-methylbutanoate | 0.33 | 0.00 | 0.00 | 0.00 |
| methyl-(2R)-2-hydroxy-3-methylbutanoate | 0.33 | 0.00 | 0.00 | 0.00 |
| methyl-(2R)-2-hydroxy-4-methylpentanoate | 0.33 | 0.00 | 0.00 | 0.00 |
| methyl-(2R,3S)-2-acetoxy-3-methylpentanoate | 0.33 | 0.00 | 0.00 | 0.00 |
| methyl-(2S,3S)-2-acetoxy-3-methylpentanoate | 0.33 | 0.00 | 0.00 | 0.00 |
| methyl 2-methylbutyrate | 0.33 | 0.00 | 0.00 | 0.00 |
| methyl 2-methylpropanoate | 0.33 | 0.00 | 0.00 | 0.00 |
| methyl 2-methylpropenoate | 0.33 | 0.00 | 0.00 | 0.00 |
| methyl 3-methylbutanoate | 0.33 | 0.00 | 0.00 | 0.00 |
| methyl 3-methylbutyrate | 0.33 | 0.00 | 0.00 | 0.00 |
| methyl 3-methylpentanoate | 0.33 | 0.00 | 0.00 | 0.00 |
| methyl 4-methylpentanoate | 0.33 | 0.00 | 0.00 | 0.00 |
| methyl caprate | 0.33 | 0.00 | 0.00 | 0.00 |
| methyl crotonate | 0.33 | 0.00 | 0.00 | 0.00 |
| methyl dodecenoate | 0.33 | 0.00 | 0.00 | 0.00 |
| methyl hydroxypentanoate | 0.33 | 0.00 | 0.00 | 0.00 |
| methyl tetradecanoate | 0.33 | 0.00 | 0.00 | 0.00 |
| methyl tetradecenoate | 0.33 | 0.00 | 0.00 | 0.00 |
| n-butyl isobutanoate | 0.33 | 0.00 | 0.00 | 0.00 |
| n-butyl tiglate | 0.33 | 0.00 | 0.00 | 0.00 |
| n-dodecane | 0.33 | 0.00 | 0.00 | 0.00 |
| n-heptadecane | 0.33 | 0.00 | 0.00 | 0.00 |
| n-heptyl acetate | 0.33 | 0.00 | 0.00 | 0.00 |
| n-hexadecane | 0.33 | 0.00 | 0.00 | 0.00 |
| n-hexyl butyrate | 0.33 | 0.00 | 0.00 | 0.00 |
| nonadecene | 0.33 | 0.00 | 0.00 | 0.00 |
| nonyl acetate | 0.33 | 0.00 | 0.00 | 0.00 |
| octadecene | 0.33 | 0.00 | 0.00 | 0.00 |
| octadecyl acetate | 0.33 | 0.00 | 0.00 | 0.00 |
| octan-3-ol | 0.33 | 0.00 | 0.00 | 0.00 |
| octane | 0.33 | 0.00 | 0.00 | 0.00 |
| octanoic acid ethyl ester | 0.33 | 0.00 | 0.00 | 0.00 |
| pent-3-yl acetate | 0.33 | 0.00 | 0.00 | 0.00 |
| pentanal | 0.33 | 0.00 | 0.00 | 0.00 |
| phenylpropyl acetate | 0.33 | 0.00 | 0.00 | 0.00 |
| prenyl acetate | 0.33 | 0.00 | 0.00 | 0.00 |
| prenyl ethanoate | 0.33 | 0.00 | 0.00 | 0.00 |
| propyl (E)-2-methyl-2-butenoate | 0.33 | 0.00 | 0.00 | 0.00 |
| propyl butanoate | 0.33 | 0.00 | 0.00 | 0.00 |
| tridecanal | 0.33 | 0.00 | 0.00 | 0.00 |
| undecanal | 0.33 | 0.00 | 0.00 | 0.00 |
| undecyl acetate | 0.33 | 0.00 | 0.00 | 0.00 |
| Z-11-hexadecenoic acid | 0.33 | 0.00 | 0.00 | 0.00 |
| δ-decalactone | 0.33 | 0.00 | 0.00 | 0.00 |
| **AMINOACID DERIVATIVES** | **9.18** | **0.66** | **0.66** | **0.33** |
| 3-methylbutyl acetate | 2.95 | 0.33 | 0.00 | 0.00 |
| 3-methyl-1-butanol | 2.62 | 0.00 | 0.00 | 0.00 |
| 2-methyl-1-butanol | 1.97 | 0.00 | 0.00 | 0.00 |
| methyl 2-methyl-2(E)-butenoate | 1.31 | 0.00 | 0.00 | 0.00 |
| methyl 2-methylbutanoate | 0.98 | 0.00 | 0.00 | 0.00 |
| isobutyl acetate | 0.66 | 0.33 | 0.33 | 0.33 |
| 2-methyl-2-buten-1-ol | 0.33 | 0.00 | 0.00 | 0.00 |
| 2-methylbutanoic acid | 0.33 | 0.00 | 0.00 | 0.00 |
| 2-methylbutyl isobutyrate | 0.33 | 0.00 | 0.00 | 0.00 |
| 2-methylbutyl tiglate | 0.33 | 0.00 | 0.00 | 0.00 |
| 2-methylpropanal | 0.33 | 0.00 | 0.00 | 0.00 |
| 3-methyl-1-butanone | 0.33 | 0.00 | 0.00 | 0.00 |
| 3-methyl-2-butenyl 3-methyl-2-butenoate | 0.33 | 0.00 | 0.00 | 0.00 |
| 3-methyl-3-buten-1-ol acetate | 0.33 | 0.00 | 0.00 | 0.00 |
| 3-methylbutanal | 0.33 | 0.00 | 0.00 | 0.00 |
| 3-methylbutanoic acid | 0.33 | 0.00 | 0.00 | 0.00 |
| 3-methylbutyl 3-methylbutanoate | 0.33 | 0.00 | 0.00 | 0.00 |
| ethyl isobutanoate | 0.33 | 0.00 | 0.00 | 0.00 |
| methyl 2-methyl-2(Z)-butenoate | 0.33 | 0.00 | 0.00 | 0.00 |
| **BENZENOIDS** | **80.66** | **41.31** | **29.84** | **18.69** |
| benzaldehyde | 55.08 | 12.46 | 3.61 | 0.98 |
| benzyl alcohol | 41.31 | 1.97 | 0.33 | 0.00 |
| methyl benzoate | 36.39 | 5.90 | 2.95 | 1.31 |
| 2-phenylethan-1-ol | 33.44 | 0.98 | 0.66 | 0.00 |
| benzyl acetate | 27.21 | 4.26 | 0.98 | 0.00 |
| methyl salicylate | 27.21 | 0.66 | 0.00 | 0.00 |
| phenylacetaldehyde | 25.90 | 7.21 | 2.62 | 0.33 |
| benzyl benzoate | 21.31 | 0.00 | 0.00 | 0.00 |
| 2-phenylethyl acetate | 11.15 | 0.33 | 0.33 | 0.00 |
| p-xylene | 8.85 | 0.33 | 0.00 | 0.00 |
| 1-ethyl-2-methyl-benzene | 8.52 | 0.00 | 0.00 | 0.00 |
| trimethylbenzene | 8.52 | 0.00 | 0.00 | 0.00 |
| p-cymene | 7.54 | 0.33 | 0.00 | 0.00 |
| phenyl benzoate | 7.54 | 0.00 | 0.00 | 0.00 |
| eugenol | 7.21 | 0.00 | 0.00 | 0.00 |
| ethyl benzoate | 6.89 | 0.00 | 0.00 | 0.00 |
| anisole | 6.23 | 1.64 | 0.33 | 0.00 |
| 1-ethyl-2,3-dimethyl-benzene | 6.23 | 0.00 | 0.00 | 0.00 |
| acetophenone | 5.90 | 0.66 | 0.33 | 0.00 |
| 1,2,3-trimethyl-benzene | 5.90 | 0.00 | 0.00 | 0.00 |
| cis-3-hexenyl benzoate | 5.90 | 0.00 | 0.00 | 0.00 |
| dimethyl salicylate | 5.57 | 0.00 | 0.00 | 0.00 |
| ethenylbenzene | 5.57 | 0.00 | 0.00 | 0.00 |
| 1,2-dimethylbenzene | 4.92 | 0.00 | 0.00 | 0.00 |
| 2,6-dimethylbenzaldehyde | 4.59 | 0.00 | 0.00 | 0.00 |
| benzoic acid | 4.59 | 0.00 | 0.00 | 0.00 |
| isoeugenol | 4.59 | 0.00 | 0.00 | 0.00 |
| methyleugenol | 4.59 | 0.00 | 0.00 | 0.00 |
| 1,4-dimethoxybenzene | 4.26 | 0.66 | 0.00 | 0.00 |
| 4-methoxybenzaldehyde | 4.26 | 0.33 | 0.00 | 0.00 |
| 1,2-dimethoxybenzene | 3.93 | 0.66 | 0.33 | 0.00 |
| styrene | 3.93 | 0.66 | 0.00 | 0.00 |
| methyl 2-hydroxybenzoate | 3.93 | 0.33 | 0.33 | 0.00 |
| butyl benzoate | 3.93 | 0.00 | 0.00 | 0.00 |
| cinnamyl alcohol | 3.93 | 0.00 | 0.00 | 0.00 |
| pentyl benzoate | 3.93 | 0.00 | 0.00 | 0.00 |
| 2-methoxy phenol | 3.61 | 0.00 | 0.00 | 0.00 |
| methyl cinnamate | 3.61 | 0.00 | 0.00 | 0.00 |
| p-cresol | 3.28 | 0.33 | 0.00 | 0.00 |
| cinnamyl acetate | 2.95 | 0.00 | 0.00 | 0.00 |
| cyclohexyl bezoate | 2.95 | 0.00 | 0.00 | 0.00 |
| trans-cinnamaldehyde | 2.95 | 0.00 | 0.00 | 0.00 |
| trans-cinnamic aldehyde | 2.95 | 0.00 | 0.00 | 0.00 |
| 4-methoxybenzyl alcohol | 2.62 | 0.00 | 0.00 | 0.00 |
| benzyl tiglate | 2.62 | 0.00 | 0.00 | 0.00 |
| isobutyl benzoate | 2.62 | 0.00 | 0.00 | 0.00 |
| trans-cinnamyl alcohol | 2.62 | 0.00 | 0.00 | 0.00 |
| 3-phenylpropanol | 2.30 | 0.00 | 0.00 | 0.00 |
| 3-phenylpropyl acetate | 2.30 | 0.00 | 0.00 | 0.00 |
| allyl benzoate | 2.30 | 0.00 | 0.00 | 0.00 |
| benzyl 3-methylbutanoate | 2.30 | 0.00 | 0.00 | 0.00 |
| benzyl butanoate | 2.30 | 0.00 | 0.00 | 0.00 |
| salicylaldehyde | 2.30 | 0.00 | 0.00 | 0.00 |
| benzenecarboxylic acid | 1.97 | 0.33 | 0.00 | 0.00 |
| benzyl isovalerate | 1.97 | 0.00 | 0.00 | 0.00 |
| cis-cinnamic aldehyde | 1.97 | 0.00 | 0.00 | 0.00 |
| hexyl benzoate | 1.97 | 0.00 | 0.00 | 0.00 |
| o-xylene | 1.97 | 0.00 | 0.00 | 0.00 |
| benzyl methyl ether | 1.64 | 0.00 | 0.00 | 0.00 |
| benzyl salicylate | 1.64 | 0.00 | 0.00 | 0.00 |
| cinnamic aldehyde (a) | 1.64 | 0.00 | 0.00 | 0.00 |
| elemicine | 1.64 | 0.00 | 0.00 | 0.00 |
| ethylbenzene | 1.64 | 0.00 | 0.00 | 0.00 |
| methyl 2-methoxybenzoate | 1.64 | 0.00 | 0.00 | 0.00 |
| methylbenzene | 1.64 | 0.00 | 0.00 | 0.00 |
| phenethyl acetate | 1.64 | 0.00 | 0.00 | 0.00 |
| 3,5-dimethoxytoluene | 1.31 | 0.33 | 0.00 | 0.00 |
| 1,2,4-trimethoxybenzene | 1.31 | 0.00 | 0.00 | 0.00 |
| 2-(4-methoxyphenyl) ethanol | 1.31 | 0.00 | 0.00 | 0.00 |
| (E)-isoeugenol | 1.31 | 0.00 | 0.00 | 0.00 |
| benzyl formate | 1.31 | 0.00 | 0.00 | 0.00 |
| methyl isoeugenol | 1.31 | 0.00 | 0.00 | 0.00 |
| phenol | 1.31 | 0.00 | 0.00 | 0.00 |
| toluene | 1.31 | 0.00 | 0.00 | 0.00 |
| vanilline | 1.31 | 0.00 | 0.00 | 0.00 |
| 1,3,5-trimethoxybenzene | 0.98 | 0.33 | 0.00 | 0.00 |
| 1-methoxy-4-methylbenzene | 0.98 | 0.00 | 0.00 | 0.00 |
| 1-phenyl-1,2-propandione | 0.98 | 0.00 | 0.00 | 0.00 |
| 2-phenoxyethanol | 0.98 | 0.00 | 0.00 | 0.00 |
| 2-phenylethyl benzoate | 0.98 | 0.00 | 0.00 | 0.00 |
| benzenepropanol | 0.98 | 0.00 | 0.00 | 0.00 |
| benzyl 2-methylbutanoate | 0.98 | 0.00 | 0.00 | 0.00 |
| benzyl 2-methylbutyrate | 0.98 | 0.00 | 0.00 | 0.00 |
| benzyl butyrate | 0.98 | 0.00 | 0.00 | 0.00 |
| benzyl valerate | 0.98 | 0.00 | 0.00 | 0.00 |
| cinnamic aldehyde (b) | 0.98 | 0.00 | 0.00 | 0.00 |
| cis-asarone | 0.98 | 0.00 | 0.00 | 0.00 |
| guaiacol | 0.98 | 0.00 | 0.00 | 0.00 |
| methyl 4-methoxybenzoate | 0.98 | 0.00 | 0.00 | 0.00 |
| phenyl acetate | 0.98 | 0.00 | 0.00 | 0.00 |
| phenylpropyl alcohol | 0.98 | 0.00 | 0.00 | 0.00 |
| 1,2,4-trimethyl-benzene | 0.66 | 0.33 | 0.00 | 0.00 |
| 1,2-dimethoxy-4-(2-propenyl) benzene | 0.66 | 0.00 | 0.00 | 0.00 |
| 1,2,3-trimethoxybenzene | 0.66 | 0.00 | 0.00 | 0.00 |
| 1,3-dimethoxy-5-methylbenzene | 0.66 | 0.00 | 0.00 | 0.00 |
| 1,4-diethyl benzene | 0.66 | 0.00 | 0.00 | 0.00 |
| 2-methoxy benzaldehyde | 0.66 | 0.00 | 0.00 | 0.00 |
| 3-methyl-2-butenyl benzoate | 0.66 | 0.00 | 0.00 | 0.00 |
| 3-methyl-3-butenyl benzoate | 0.66 | 0.00 | 0.00 | 0.00 |
| 3-methylbutyl benzoate | 0.66 | 0.00 | 0.00 | 0.00 |
| 3-phenyl-1-propanol acetate | 0.66 | 0.00 | 0.00 | 0.00 |
| 4-hydroxy-3-methoxybenzaldehyde | 0.66 | 0.00 | 0.00 | 0.00 |
| 4-methylanisole | 0.66 | 0.00 | 0.00 | 0.00 |
| 4-methylphenol | 0.66 | 0.00 | 0.00 | 0.00 |
| (E)-methyl isoeugenol | 0.66 | 0.00 | 0.00 | 0.00 |
| (Z)-3-hexenyl benzoate | 0.66 | 0.00 | 0.00 | 0.00 |
| (Z)-isoeugenol + vanilline | 0.66 | 0.00 | 0.00 | 0.00 |
| (Z)-methyl isoeugenol | 0.66 | 0.00 | 0.00 | 0.00 |
| acetyl eugenol | 0.66 | 0.00 | 0.00 | 0.00 |
| amino benzaldehyde | 0.66 | 0.00 | 0.00 | 0.00 |
| benzenepropanal | 0.66 | 0.00 | 0.00 | 0.00 |
| benzyl 2-hydroxybenzoate | 0.66 | 0.00 | 0.00 | 0.00 |
| benzyl ester | 0.66 | 0.00 | 0.00 | 0.00 |
| benzyl propanate | 0.66 | 0.00 | 0.00 | 0.00 |
| estragol | 0.66 | 0.00 | 0.00 | 0.00 |
| isoelemicine | 0.66 | 0.00 | 0.00 | 0.00 |
| methyl chavicol | 0.66 | 0.00 | 0.00 | 0.00 |
| o-cymene | 0.66 | 0.00 | 0.00 | 0.00 |
| phenylpropyl acetate | 0.66 | 0.00 | 0.00 | 0.00 |
| propyl benzoate | 0.66 | 0.00 | 0.00 | 0.00 |
| styrol | 0.66 | 0.00 | 0.00 | 0.00 |
| trans-asarone | 0.66 | 0.00 | 0.00 | 0.00 |
| trans-cinnamyl acetate | 0.66 | 0.00 | 0.00 | 0.00 |
| trans-ethyl cinnamate | 0.66 | 0.00 | 0.00 | 0.00 |
| trans-methyl cinnamate | 0.66 | 0.00 | 0.00 | 0.00 |
| trimethoxybenzene | 0.66 | 0.00 | 0.00 | 0.00 |
| veratrole | 0.66 | 0.00 | 0.00 | 0.00 |
| benzoic acid methyl ester | 0.33 | 0.33 | 0.33 | 0.00 |
| 1hydroxy-1-phenyl-2-propanone | 0.33 | 0.00 | 0.00 | 0.00 |
| 1-methoxy-4-vinylbenzene | 0.33 | 0.00 | 0.00 | 0.00 |
| 1-methyl-4-(1methylethenyl)-benzene | 0.33 | 0.00 | 0.00 | 0.00 |
| 1-methylphenol | 0.33 | 0.00 | 0.00 | 0.00 |
| 1-phenyl-2,3-butandione | 0.33 | 0.00 | 0.00 | 0.00 |
| 1,2-benzenedicarboxylic acid | 0.33 | 0.00 | 0.00 | 0.00 |
| 1,2-dimethoxy-4-methylbenzene | 0.33 | 0.00 | 0.00 | 0.00 |
| 1,2,3-trimethoxy-5-methylbenzene | 0.33 | 0.00 | 0.00 | 0.00 |
| 1,2,3,4-tetramethyl-benzene | 0.33 | 0.00 | 0.00 | 0.00 |
| 1,3-dimethoxybenzene | 0.33 | 0.00 | 0.00 | 0.00 |
| 1,3,5-trimethyl-benzene | 0.33 | 0.00 | 0.00 | 0.00 |
| 2-(4-methoxyphenyl) ethanol | 0.33 | 0.00 | 0.00 | 0.00 |
| 2-amino benzene thiol | 0.33 | 0.00 | 0.00 | 0.00 |
| 2-amino benzoic acid ethyl ester | 0.33 | 0.00 | 0.00 | 0.00 |
| 2-methoxy p-cresol | 0.33 | 0.00 | 0.00 | 0.00 |
| 2-methoxybenzyl acetate | 0.33 | 0.00 | 0.00 | 0.00 |
| 2-methoxybenzyl alcohol | 0.33 | 0.00 | 0.00 | 0.00 |
| 2-methyl benzaldehyde | 0.33 | 0.00 | 0.00 | 0.00 |
| 2-phenylacetonitrile | 0.33 | 0.00 | 0.00 | 0.00 |
| 2-phenylethyl 2-methylpropanoate | 0.33 | 0.00 | 0.00 | 0.00 |
| 2-phenylpropenal | 0.33 | 0.00 | 0.00 | 0.00 |
| 2,6-dimethoxy-4-(2-propenyl)-phenol | 0.33 | 0.00 | 0.00 | 0.00 |
| 3-ethylbenzaldehyde | 0.33 | 0.00 | 0.00 | 0.00 |
| 3-phenylpropyl alcohol | 0.33 | 0.00 | 0.00 | 0.00 |
| 4-hydroxybenzaldehyde | 0.33 | 0.00 | 0.00 | 0.00 |
| 4-methoxybenzyl acetate | 0.33 | 0.00 | 0.00 | 0.00 |
| 4-methoxycinnamic aldehyde | 0.33 | 0.00 | 0.00 | 0.00 |
| 4-methoxymethyl benzoate | 0.33 | 0.00 | 0.00 | 0.00 |
| 4-methoxyphenol | 0.33 | 0.00 | 0.00 | 0.00 |
| 4-methoxy phenyl ethyl alcohol | 0.33 | 0.00 | 0.00 | 0.00 |
| 4-methoxyphenyl propanol | 0.33 | 0.00 | 0.00 | 0.00 |
| 4-methoxytoluene | 0.33 | 0.00 | 0.00 | 0.00 |
| 4-methylguaiacol | 0.33 | 0.00 | 0.00 | 0.00 |
| 4-phenyl-3-buten-2-one | 0.33 | 0.00 | 0.00 | 0.00 |
| anetole | 0.33 | 0.00 | 0.00 | 0.00 |
| anisaldehyde | 0.33 | 0.00 | 0.00 | 0.00 |
| anisyl acetate | 0.33 | 0.00 | 0.00 | 0.00 |
| arasole | 0.33 | 0.00 | 0.00 | 0.00 |
| benzenepropyl acetate | 0.33 | 0.00 | 0.00 | 0.00 |
| benzoic acid ethyl ester | 0.33 | 0.00 | 0.00 | 0.00 |
| benzyl 2-methylpropanoate | 0.33 | 0.00 | 0.00 | 0.00 |
| benzyl benzoic acid | 0.33 | 0.00 | 0.00 | 0.00 |
| benzyl iso-pentanoate | 0.33 | 0.00 | 0.00 | 0.00 |
| benzyl pentanoate | 0.33 | 0.00 | 0.00 | 0.00 |
| benzyl propionate | 0.33 | 0.00 | 0.00 | 0.00 |
| carvacrol | 0.33 | 0.00 | 0.00 | 0.00 |
| chavicol | 0.33 | 0.00 | 0.00 | 0.00 |
| cinnamyl alcohol acetate | 0.33 | 0.00 | 0.00 | 0.00 |
| cis-cinnamadehyde | 0.33 | 0.00 | 0.00 | 0.00 |
| ethyl 3-phenylpropanoate | 0.33 | 0.00 | 0.00 | 0.00 |
| ethyl cinnamate | 0.33 | 0.00 | 0.00 | 0.00 |
| ethyl phenylacetate | 0.33 | 0.00 | 0.00 | 0.00 |
| eugenol acetate | 0.33 | 0.00 | 0.00 | 0.00 |
| hemimelitene | 0.33 | 0.00 | 0.00 | 0.00 |
| isobutyl 2-hydroxibenzoate | 0.33 | 0.00 | 0.00 | 0.00 |
| isobutyl phenylacetate | 0.33 | 0.00 | 0.00 | 0.00 |
| isopentyl benzoate | 0.33 | 0.00 | 0.00 | 0.00 |
| isopentyl 2-hydroxibenzoate | 0.33 | 0.00 | 0.00 | 0.00 |
| isopropyl salicylate | 0.33 | 0.00 | 0.00 | 0.00 |
| mesitylene | 0.33 | 0.00 | 0.00 | 0.00 |
| methyl 2-hydroxy-3-phenylpropanoate | 0.33 | 0.00 | 0.00 | 0.00 |
| methyl 2-phenylacetate | 0.33 | 0.00 | 0.00 | 0.00 |
| methyl 2,3-dimethoxybenzoate | 0.33 | 0.00 | 0.00 | 0.00 |
| methyl 3-phenylpropanoate | 0.33 | 0.00 | 0.00 | 0.00 |
| methyl 3,5-dimethoxybenzoate | 0.33 | 0.00 | 0.00 | 0.00 |
| methyl 4-methoxysalicylate | 0.33 | 0.00 | 0.00 | 0.00 |
| methyl antranilate | 0.33 | 0.00 | 0.00 | 0.00 |
| methyl mandelate | 0.33 | 0.00 | 0.00 | 0.00 |
| methyl p-anisate | 0.33 | 0.00 | 0.00 | 0.00 |
| n-butyl benzoate | 0.33 | 0.00 | 0.00 | 0.00 |
| n-hexenyl benzoate | 0.33 | 0.00 | 0.00 | 0.00 |
| n-phenylformamide | 0.33 | 0.00 | 0.00 | 0.00 |
| p-anisyl alcohol | 0.33 | 0.00 | 0.00 | 0.00 |
| p-propenylanisole | 0.33 | 0.00 | 0.00 | 0.00 |
| pentyl salicylate | 0.33 | 0.00 | 0.00 | 0.00 |
| phenethyl benzoate | 0.33 | 0.00 | 0.00 | 0.00 |
| phenylethyl tiglate | 0.33 | 0.00 | 0.00 | 0.00 |
| phenylpropanal | 0.33 | 0.00 | 0.00 | 0.00 |
| phenylpropanol | 0.33 | 0.00 | 0.00 | 0.00 |
| propiophenone | 0.33 | 0.00 | 0.00 | 0.00 |
| propylbenzene | 0.33 | 0.00 | 0.00 | 0.00 |
| vanilline methylketone | 0.33 | 0.00 | 0.00 | 0.00 |
| **TOTAL TERPENES** | **88.20** | **54.75** | **41.64** | **26.23** |
| **MONOTERPENES** | **80.33** | **41.31** | **29.18** | **14.75** |
| limonene | 48.20 | 1.97 | 0.33 | 0.33 |
| trans-β-ocimene | 45.25 | 10.16 | 4.92 | 0.66 |
| linalool | 44.59 | 9.18 | 4.92 | 1.31 |
| α-pinene | 33.77 | 2.62 | 0.66 | 0.33 |
| cis-β-ocimene | 26.23 | 1.31 | 0.33 | 0.00 |
| β-pinene | 24.92 | 0.33 | 0.00 | 0.00 |
| myrcene | 19.02 | 0.33 | 0.00 | 0.00 |
| eucalyptol | 14.10 | 2.62 | 1.31 | 0.00 |
| cis-linalool oxide (furanoid) | 12.46 | 0.00 | 0.00 | 0.00 |
| β-myrcene | 11.80 | 0.33 | 0.00 | 0.00 |
| sabinene | 11.80 | 0.00 | 0.00 | 0.00 |
| α-terpineol | 11.15 | 0.33 | 0.00 | 0.00 |
| geranylacetone | 10.16 | 0.00 | 0.00 | 0.00 |
| trans-linalool oxide (furanoid) | 9.84 | 0.00 | 0.00 | 0.00 |
| 3-carene | 8.52 | 0.66 | 0.00 | 0.00 |
| camphene | 8.20 | 0.33 | 0.00 | 0.00 |
| linalool oxide (pyranoid) I | 8.20 | 0.00 | 0.00 | 0.00 |
| lilac aldehyde A | 6.56 | 0.98 | 0.00 | 0.00 |
| nerol | 6.23 | 0.33 | 0.00 | 0.00 |
| lilac aldehyde B | 5.90 | 0.33 | 0.00 | 0.00 |
| α-terpinolene | 5.90 | 0.00 | 0.00 | 0.00 |
| γ-terpinene | 5.57 | 0.33 | 0.00 | 0.00 |
| α-phellandrene | 5.25 | 0.00 | 0.00 | 0.00 |
| lilac aldehyde D | 4.92 | 0.00 | 0.00 | 0.00 |
| geraniol | 4.59 | 0.00 | 0.00 | 0.00 |
| trans-ocimenol | 4.59 | 0.00 | 0.00 | 0.00 |
| allo-ocimene | 4.26 | 0.00 | 0.00 | 0.00 |
| camphor | 4.26 | 0.00 | 0.00 | 0.00 |
| cis-geranyl acetone | 4.26 | 0.00 | 0.00 | 0.00 |
| lilac aldehyde C | 4.26 | 0.00 | 0.00 | 0.00 |
| β-phellandrene | 4.26 | 0.00 | 0.00 | 0.00 |
| linalool oxide (pyranoid) II | 3.61 | 0.00 | 0.00 | 0.00 |
| terpinolene | 3.61 | 0.00 | 0.00 | 0.00 |
| citronellol | 3.28 | 0.33 | 0.00 | 0.00 |
| lilac alcohol A | 3.28 | 0.00 | 0.00 | 0.00 |
| lilac alcohol D | 2.95 | 0.00 | 0.00 | 0.00 |
| linalool oxide | 2.95 | 0.00 | 0.00 | 0.00 |
| trans-β-ocimene epoxide | 2.95 | 0.00 | 0.00 | 0.00 |
| geranial | 2.62 | 0.00 | 0.00 | 0.00 |
| α-terpinene | 2.62 | 0.00 | 0.00 | 0.00 |
| geranyl acetate | 2.30 | 0.00 | 0.00 | 0.00 |
| hotrienol | 2.30 | 0.00 | 0.00 | 0.00 |
| neral | 2.30 | 0.00 | 0.00 | 0.00 |
| verbenone | 2.30 | 0.00 | 0.00 | 0.00 |
| 4-terpineol | 1.97 | 0.00 | 0.00 | 0.00 |
| (E,E)-2,6-dimethyl-1,3,5,7-ocatetraene | 1.97 | 0.00 | 0.00 | 0.00 |
| (E,Z)-2,6-dimethyl-1,3,5,7-octatetraene | 1.97 | 0.00 | 0.00 | 0.00 |
| lavandulol | 1.97 | 0.00 | 0.00 | 0.00 |
| lilac alcohol | 1.97 | 0.00 | 0.00 | 0.00 |
| lilac alcohol B | 1.97 | 0.00 | 0.00 | 0.00 |
| 2,6-dimethyl-3,7-octadiene-2,6-diol | 1.64 | 0.00 | 0.00 | 0.00 |
| carveol | 1.64 | 0.00 | 0.00 | 0.00 |
| cis-citral | 1.64 | 0.00 | 0.00 | 0.00 |
| citronellal | 1.64 | 0.00 | 0.00 | 0.00 |
| lilac alcohol C | 1.64 | 0.00 | 0.00 | 0.00 |
| limonene diepoxides | 1.64 | 0.00 | 0.00 | 0.00 |
| lilac aldehyde B + C | 1.31 | 0.33 | 0.00 | 0.00 |
| 6-ethenyltetrahydro-2,2,6-trimethyl-2H-pyran-3(4H)-one | 1.31 | 0.00 | 0.00 | 0.00 |
| (E)-3,7-dimethyl-2,6-octadien-1-ol | 1.31 | 0.00 | 0.00 | 0.00 |
| ipsdienone | 1.31 | 0.00 | 0.00 | 0.00 |
| menthatriene isomer 5 | 1.31 | 0.00 | 0.00 | 0.00 |
| perillene | 1.31 | 0.00 | 0.00 | 0.00 |
| pinocarvone | 1.31 | 0.00 | 0.00 | 0.00 |
| thymol | 1.31 | 0.00 | 0.00 | 0.00 |
| α-thujene | 1.31 | 0.00 | 0.00 | 0.00 |
| lilac alcohol B + C | 0.98 | 0.33 | 0.00 | 0.00 |
| (Z)-3,7-dimethyl-2,6-octadien-1-ol | 0.98 | 0.00 | 0.00 | 0.00 |
| bornyl acetate | 0.98 | 0.00 | 0.00 | 0.00 |
| citronellyl acetate | 0.98 | 0.00 | 0.00 | 0.00 |
| dihydrocarvone | 0.98 | 0.00 | 0.00 | 0.00 |
| lilac alcohol formate | 0.98 | 0.00 | 0.00 | 0.00 |
| linalool oxide (pyranoid: alcohol) | 0.98 | 0.00 | 0.00 | 0.00 |
| menthatriene | 0.98 | 0.00 | 0.00 | 0.00 |
| menthol | 0.98 | 0.00 | 0.00 | 0.00 |
| methyl geranate | 0.98 | 0.00 | 0.00 | 0.00 |
| thujol | 0.98 | 0.00 | 0.00 | 0.00 |
| trans-geranyl acetone | 0.98 | 0.00 | 0.00 | 0.00 |
| ipsdienol | 0.66 | 0.33 | 0.33 | 0.00 |
| neryl acetate | 0.66 | 0.33 | 0.33 | 0.00 |
| ocimene | 0.66 | 0.33 | 0.00 | 0.00 |
| 2,6-dimethyl-1,7-octadiene-3,6-diol | 0.66 | 0.00 | 0.00 | 0.00 |
| (1R)-(-)-myrtenal | 0.66 | 0.00 | 0.00 | 0.00 |
| borneol | 0.66 | 0.00 | 0.00 | 0.00 |
| carvone | 0.66 | 0.00 | 0.00 | 0.00 |
| cis-carveole | 0.66 | 0.00 | 0.00 | 0.00 |
| cis-geraniol | 0.66 | 0.00 | 0.00 | 0.00 |
| cis-sabinene hydrate | 0.66 | 0.00 | 0.00 | 0.00 |
| isocitronellene | 0.66 | 0.00 | 0.00 | 0.00 |
| lavandulyl acetate | 0.66 | 0.00 | 0.00 | 0.00 |
| lilac acetate A | 0.66 | 0.00 | 0.00 | 0.00 |
| linalyl acetate | 0.66 | 0.00 | 0.00 | 0.00 |
| menthatriene isomer 3 | 0.66 | 0.00 | 0.00 | 0.00 |
| menthatriene isomer 4 | 0.66 | 0.00 | 0.00 | 0.00 |
| myrcene epoxide | 0.66 | 0.00 | 0.00 | 0.00 |
| myrtenol | 0.66 | 0.00 | 0.00 | 0.00 |
| octadiene | 0.66 | 0.00 | 0.00 | 0.00 |
| sabinene + α-pinene | 0.66 | 0.00 | 0.00 | 0.00 |
| trans-4-thujanol | 0.66 | 0.00 | 0.00 | 0.00 |
| trans-citral | 0.66 | 0.00 | 0.00 | 0.00 |
| trans-geraniol | 0.66 | 0.00 | 0.00 | 0.00 |
| tricyclene | 0.66 | 0.00 | 0.00 | 0.00 |
| α-ocimene | 0.66 | 0.00 | 0.00 | 0.00 |
| β-terpinene | 0.66 | 0.00 | 0.00 | 0.00 |
| geranyl methyl ether | 0.33 | 0.33 | 0.33 | 0.33 |
| 1,2-dimethoxybenzene | 0.33 | 0.00 | 0.00 | 0.00 |
| 2-carene | 0.33 | 0.00 | 0.00 | 0.00 |
| 2,3,6-trimethyl-1,5-heptadiene | 0.33 | 0.00 | 0.00 | 0.00 |
| 2,7-methyl octadiene | 0.33 | 0.00 | 0.00 | 0.00 |
| 3-methyl-6-(-1-methylethyl)-2-cyclohexen-1-one | 0.33 | 0.00 | 0.00 | 0.00 |
| 3,6,6-trimethyl-bicyclo [3.1.1] heptan-2-one | 0.33 | 0.00 | 0.00 | 0.00 |
| 5-ethenyldihydro-5-methyl-2(3H)-furanone | 0.33 | 0.00 | 0.00 | 0.00 |
| 5-hydroxycineole | 0.33 | 0.00 | 0.00 | 0.00 |
| (E,E)-neo-allo-ocimene | 0.33 | 0.00 | 0.00 | 0.00 |
| (E,Z)-2,6-dimethyl-2,5,7-octatrien-1-ol | 0.33 | 0.00 | 0.00 | 0.00 |
| (E,Z)-allo-ocimene | 0.33 | 0.00 | 0.00 | 0.00 |
| amitinol | 0.33 | 0.00 | 0.00 | 0.00 |
| camphene epoxide 1 | 0.33 | 0.00 | 0.00 | 0.00 |
| camphene epoxide 2 | 0.33 | 0.00 | 0.00 | 0.00 |
| chrysanthenone | 0.33 | 0.00 | 0.00 | 0.00 |
| cis-4-thujanol | 0.33 | 0.00 | 0.00 | 0.00 |
| cis-ocimenone | 0.33 | 0.00 | 0.00 | 0.00 |
| cis-rose oxide | 0.33 | 0.00 | 0.00 | 0.00 |
| cis-β-ocimene epoxide | 0.33 | 0.00 | 0.00 | 0.00 |
| citronellic acid methyl ester | 0.33 | 0.00 | 0.00 | 0.00 |
| citronellyl formate | 0.33 | 0.00 | 0.00 | 0.00 |
| eucarvone | 0.33 | 0.00 | 0.00 | 0.00 |
| exo-2-hydroxycineole | 0.33 | 0.00 | 0.00 | 0.00 |
| fenchol | 0.33 | 0.00 | 0.00 | 0.00 |
| fenchyl acetate | 0.33 | 0.00 | 0.00 | 0.00 |
| geranic acid methyl ester | 0.33 | 0.00 | 0.00 | 0.00 |
| geranyl propanoate | 0.33 | 0.00 | 0.00 | 0.00 |
| ipsenone | 0.33 | 0.00 | 0.00 | 0.00 |
| isoborneol | 0.33 | 0.00 | 0.00 | 0.00 |
| isobornyl acetate | 0.33 | 0.00 | 0.00 | 0.00 |
| isopinocamphone | 0.33 | 0.00 | 0.00 | 0.00 |
| lavandulyl propanoate | 0.33 | 0.00 | 0.00 | 0.00 |
| lilac acetate B | 0.33 | 0.00 | 0.00 | 0.00 |
| lilac acetate C | 0.33 | 0.00 | 0.00 | 0.00 |
| lilac acetate D | 0.33 | 0.00 | 0.00 | 0.00 |
| lilac aldehyde | 0.33 | 0.00 | 0.00 | 0.00 |
| lilac degradation | 0.33 | 0.00 | 0.00 | 0.00 |
| linalool oxide acetate (pyranoid) | 0.33 | 0.00 | 0.00 | 0.00 |
| linalool oxide (pyranoid: ketone) | 0.33 | 0.00 | 0.00 | 0.00 |
| menthatriene isomer 1 | 0.33 | 0.00 | 0.00 | 0.00 |
| menthatriene isomer 2 | 0.33 | 0.00 | 0.00 | 0.00 |
| methyl geranylate | 0.33 | 0.00 | 0.00 | 0.00 |
| myrcene oxide | 0.33 | 0.00 | 0.00 | 0.00 |
| nerylacetone | 0.33 | 0.00 | 0.00 | 0.00 |
| neryl methyl ether | 0.33 | 0.00 | 0.00 | 0.00 |
| phellandrene | 0.33 | 0.00 | 0.00 | 0.00 |
| santene | 0.33 | 0.00 | 0.00 | 0.00 |
| terpenediol | 0.33 | 0.00 | 0.00 | 0.00 |
| trans-geranic acid | 0.33 | 0.00 | 0.00 | 0.00 |
| trans-ocimenone | 0.33 | 0.00 | 0.00 | 0.00 |
| trans-rose oxide | 0.33 | 0.00 | 0.00 | 0.00 |
| trans-sabinene hydrate | 0.33 | 0.00 | 0.00 | 0.00 |
| α-fenchene | 0.33 | 0.00 | 0.00 | 0.00 |
| α-terpinyl acetate | 0.33 | 0.00 | 0.00 | 0.00 |
| α-terpinyl methyl ether | 0.33 | 0.00 | 0.00 | 0.00 |
| β-citronellene | 0.33 | 0.00 | 0.00 | 0.00 |
| β-citronellol | 0.33 | 0.00 | 0.00 | 0.00 |
| **SESQUITERPENES** | **57.05** | **7.87** | **5.25** | **2.62** |
| β-caryophyllene | 16.39 | 0.66 | 0.66 | 0.00 |
| α-humulene | 13.44 | 0.00 | 0.00 | 0.00 |
| α-farnesene | 13.11 | 0.66 | 0.33 | 0.00 |
| caryophyllene | 12.46 | 0.98 | 0.98 | 0.00 |
| α-copaene | 11.15 | 0.00 | 0.00 | 0.00 |
| germacrene D | 8.52 | 0.33 | 0.00 | 0.00 |
| nerolidol | 7.21 | 0.98 | 0.66 | 0.66 |
| δ-cadinene | 5.57 | 0.00 | 0.00 | 0.00 |
| trans,trans-α-farnesene | 5.25 | 0.00 | 0.00 | 0.00 |
| valencene | 4.92 | 0.00 | 0.00 | 0.00 |
| caryophyllene oxide | 4.59 | 0.00 | 0.00 | 0.00 |
| trans-β-farnesene | 4.59 | 0.00 | 0.00 | 0.00 |
| β-bourbonene | 4.59 | 0.00 | 0.00 | 0.00 |
| trans-nerolidol | 4.26 | 0.33 | 0.33 | 0.33 |
| trans,trans-farnesol | 3.61 | 0.33 | 0.00 | 0.00 |
| γ-muurolene | 3.61 | 0.33 | 0.00 | 0.00 |
| γ-cadinene | 3.61 | 0.00 | 0.00 | 0.00 |
| α-gurjunene | 3.28 | 0.00 | 0.00 | 0.00 |
| β-elemene | 3.28 | 0.00 | 0.00 | 0.00 |
| β-farnesene | 3.28 | 0.00 | 0.00 | 0.00 |
| δ-elemene | 3.28 | 0.00 | 0.00 | 0.00 |
| β-cedrene | 2.95 | 0.00 | 0.00 | 0.00 |
| β-selinene | 2.95 | 0.00 | 0.00 | 0.00 |
| α-cubebene | 2.62 | 0.00 | 0.00 | 0.00 |
| α-muurolene | 2.62 | 0.00 | 0.00 | 0.00 |
| trans-α-bergamotene | 2.30 | 0.00 | 0.00 | 0.00 |
| α-bergamotene | 2.30 | 0.00 | 0.00 | 0.00 |
| β-copaene | 2.30 | 0.00 | 0.00 | 0.00 |
| β-cubebene | 2.30 | 0.00 | 0.00 | 0.00 |
| chamigrene | 1.97 | 0.00 | 0.00 | 0.00 |
| cis, trans-α-farnesene | 1.97 | 0.00 | 0.00 | 0.00 |
| β-sesquiphellandrene | 1.97 | 0.00 | 0.00 | 0.00 |
| γ-elemene | 1.97 | 0.00 | 0.00 | 0.00 |
| farnesol | 1.64 | 0.00 | 0.00 | 0.00 |
| trans-α-farnesene | 1.64 | 0.00 | 0.00 | 0.00 |
| 12-oxabicyclo dodecadiene | 1.31 | 0.00 | 0.00 | 0.00 |
| bicyclogermacrene | 1.31 | 0.00 | 0.00 | 0.00 |
| cis,cis-farnesol | 1.31 | 0.00 | 0.00 | 0.00 |
| α-cedrene | 1.31 | 0.00 | 0.00 | 0.00 |
| α-ylangene | 1.31 | 0.00 | 0.00 | 0.00 |
| β-gurjunene | 1.31 | 0.00 | 0.00 | 0.00 |
| allo-aromadendrene | 0.98 | 0.00 | 0.00 | 0.00 |
| cis-calamene | 0.98 | 0.00 | 0.00 | 0.00 |
| cis-nerolidol | 0.98 | 0.00 | 0.00 | 0.00 |
| cis,trans-farnesol | 0.98 | 0.00 | 0.00 | 0.00 |
| cis-β-farnesene | 0.98 | 0.00 | 0.00 | 0.00 |
| junipene | 0.98 | 0.00 | 0.00 | 0.00 |
| longifolene | 0.98 | 0.00 | 0.00 | 0.00 |
| seychellene | 0.98 | 0.00 | 0.00 | 0.00 |
| γ-gurjunene | 0.98 | 0.00 | 0.00 | 0.00 |
| δ-selinene | 0.98 | 0.00 | 0.00 | 0.00 |
| calamene | 0.66 | 0.00 | 0.00 | 0.00 |
| cis-α-bergamotene | 0.66 | 0.00 | 0.00 | 0.00 |
| germacrene B | 0.66 | 0.00 | 0.00 | 0.00 |
| spathulenol | 0.66 | 0.00 | 0.00 | 0.00 |
| trans,trans-farnesal | 0.66 | 0.00 | 0.00 | 0.00 |
| trans-β-ionone | 0.66 | 0.00 | 0.00 | 0.00 |
| α-selinene | 0.66 | 0.00 | 0.00 | 0.00 |
| α-zingiberene | 0.66 | 0.00 | 0.00 | 0.00 |
| α,β-eudesmol | 0.66 | 0.00 | 0.00 | 0.00 |
| β-bisabolene | 0.66 | 0.00 | 0.00 | 0.00 |
| (+)-γ-cadinene | 0.33 | 0.00 | 0.00 | 0.00 |
| (E)-β-bergamotene | 0.33 | 0.00 | 0.00 | 0.00 |
| 1,5-cyclo-undecadiene | 0.33 | 0.00 | 0.00 | 0.00 |
| AR-curcumene | 0.33 | 0.00 | 0.00 | 0.00 |
| aristolene | 0.33 | 0.00 | 0.00 | 0.00 |
| aromadendrene epoxide | 0.33 | 0.00 | 0.00 | 0.00 |
| bergamotene | 0.33 | 0.00 | 0.00 | 0.00 |
| carotol | 0.33 | 0.00 | 0.00 | 0.00 |
| cis,trans-farnesal | 0.33 | 0.00 | 0.00 | 0.00 |
| cis-β-guaiene | 0.33 | 0.00 | 0.00 | 0.00 |
| dehydrogeosmin | 0.33 | 0.00 | 0.00 | 0.00 |
| dendrolasin | 0.33 | 0.00 | 0.00 | 0.00 |
| germacrone | 0.33 | 0.00 | 0.00 | 0.00 |
| guaiol | 0.33 | 0.00 | 0.00 | 0.00 |
| longicyclene | 0.33 | 0.00 | 0.00 | 0.00 |
| nerolidol epoxyacetate | 0.33 | 0.00 | 0.00 | 0.00 |
| T-cadinol | 0.33 | 0.00 | 0.00 | 0.00 |
| T-muurolol | 0.33 | 0.00 | 0.00 | 0.00 |
| thujopsene | 0.33 | 0.00 | 0.00 | 0.00 |
| trans,trans-farnesyl acetate | 0.33 | 0.00 | 0.00 | 0.00 |
| trans-tagetone | 0.33 | 0.00 | 0.00 | 0.00 |
| trans-β-bergamotene | 0.33 | 0.00 | 0.00 | 0.00 |
| trans-β-farnesene oxide | 0.33 | 0.00 | 0.00 | 0.00 |
| viridiflorene | 0.33 | 0.00 | 0.00 | 0.00 |
| α-calacorene | 0.33 | 0.00 | 0.00 | 0.00 |
| α-guaiene | 0.33 | 0.00 | 0.00 | 0.00 |
| α-longipinene | 0.33 | 0.00 | 0.00 | 0.00 |
| β-cadinene | 0.33 | 0.00 | 0.00 | 0.00 |
| β-santalene | 0.33 | 0.00 | 0.00 | 0.00 |
| δ-cadinol | 0.33 | 0.00 | 0.00 | 0.00 |
| **IRREGULAR TERPENES** | **39.34** | **5.25** | **1.31** | **0.33** |
| 6-methyl-5-hepten-2-one | 22.30 | 0.33 | 0.00 | 0.00 |
| 4,8-dimethyl-nona-1,3,7-triene | 18.36 | 0.98 | 0.00 | 0.00 |
| oxoisophorone (2,6,6-trimethyl-2-cyclohexene-1,4-dione) | 3.93 | 0.66 | 0.00 | 0.00 |
| 4,8,12-trimethyl-1,3-(E)-7-(E)-11-tridecatetraene | 3.61 | 0.00 | 0.00 | 0.00 |
| β-ionone | 2.62 | 0.33 | 0.00 | 0.00 |
| dihydro-β-ionone | 1.31 | 0.33 | 0.33 | 0.00 |
| oxoisophoroneoxide (1,3,3-trimethyl-7-oxabicyclo[4.1.0]heptan-2,5-dione) | 1.31 | 0.33 | 0.00 | 0.00 |
| 4,8,12-trimethyl-1,3-(Z)-7-(E)-11-tridecatetraene | 1.31 | 0.00 | 0.00 | 0.00 |
| dihydrooxoisophorone | 1.31 | 0.00 | 0.00 | 0.00 |
| 2,2,6-trimethylcyclohexane-1,4-dione | 0.98 | 0.00 | 0.00 | 0.00 |
| 6-methyl-5-hepten-2-ol | 0.98 | 0.00 | 0.00 | 0.00 |
| dihydro-β-ionol | 0.66 | 0.33 | 0.00 | 0.00 |
| 1,3,4-trimethyl-3-cyclohexene-1-carboxaldehyde | 0.66 | 0.00 | 0.00 | 0.00 |
| 2,6-dimethyl-3,7-octadiene-2,6-diol | 0.66 | 0.00 | 0.00 | 0.00 |
| 4,8,12-trimethyltrideca-1,3,7,11-tetraene | 0.66 | 0.00 | 0.00 | 0.00 |
| 6,10-dimethyl-5,9-undecadien-2-ol | 0.66 | 0.00 | 0.00 | 0.00 |
| dihydro-α-ionone | 0.66 | 0.00 | 0.00 | 0.00 |
| α-ionone | 0.66 | 0.00 | 0.00 | 0.00 |
| 6,10,14-trimethylpentadecan-2-one | 0.33 | 0.33 | 0.33 | 0.00 |
| ketoisophorone | 0.33 | 0.33 | 0.00 | 0.00 |
| 2-methanol-bicyclo[3.1.1]hept-2-ene | 0.33 | 0.00 | 0.00 | 0.00 |
| 2-methyl-3-buten-2-ol | 0.33 | 0.00 | 0.00 | 0.00 |
| 2-methyl-6-methylene-1,3,7-octatriene | 0.33 | 0.00 | 0.00 | 0.00 |
| 2,6,6-trimethyl-1, 3-cyclohexadiene-1-carboxaldehyde | 0.33 | 0.00 | 0.00 | 0.00 |
| 2,6,6-trimethyl-1-cyclohexene-1-carboxaldehyde (b-cyclocitral) | 0.33 | 0.00 | 0.00 | 0.00 |
| 2,6-dimethyl-1,3,5,7-octatetraene | 0.33 | 0.00 | 0.00 | 0.00 |
| 2,6-dimethyl-3,5,7-octatriene-2-ol | 0.33 | 0.00 | 0.00 | 0.00 |
| 2,6-dimethyl-7-octen-2-ol | 0.33 | 0.00 | 0.00 | 0.00 |
| 3-methyl-3-butenol | 0.33 | 0.00 | 0.00 | 0.00 |
| 3,3-dimethyl-bicyclo[2.2.1]heptan-2-one | 0.33 | 0.00 | 0.00 | 0.00 |
| 3,4-dimethyl-2,4,6-octatriene | 0.33 | 0.00 | 0.00 | 0.00 |
| 3,5,5-trimethyl-2-cyclohexen-1-one | 0.33 | 0.00 | 0.00 | 0.00 |
| 5-methyl-3-hexene-2-one | 0.33 | 0.00 | 0.00 | 0.00 |
| 6,6-dimethyl-bicyclo[3.1.1]heptan-2-one | 0.33 | 0.00 | 0.00 | 0.00 |
| 6,7-epoxy myrcene (2,2-dimethyl-3-(methy-ene-4-penten-1-yl)-oxirane) | 0.33 | 0.00 | 0.00 | 0.00 |
| cyclic β-ionone | 0.33 | 0.00 | 0.00 | 0.00 |
| dihydroactinidiolide | 0.33 | 0.00 | 0.00 | 0.00 |
| dihydro-β-ionone epoxide | 0.33 | 0.00 | 0.00 | 0.00 |
| isophorone (3,5,5-trimethyl-3-cyclohexene-1-one) | 0.33 | 0.00 | 0.00 | 0.00 |
| tridecatetraene | 0.33 | 0.00 | 0.00 | 0.00 |
| β-cyclocitral | 0.33 | 0.00 | 0.00 | 0.00 |
| **NITROGEN CONTAINING COMPOUNDS** | **30.82** | **1.97** | **0.98** | **0.66** |
| indole | 16.72 | 0.00 | 0.00 | 0.00 |
| phenyl acetonitrile | 10.49 | 0.00 | 0.00 | 0.00 |
| methyl anthranilate | 6.56 | 0.66 | 0.33 | 0.33 |
| 2-methyl-butyl aldoxime | 3.93 | 0.33 | 0.33 | 0.00 |
| 3-methylbutyraldoxime | 3.61 | 0.33 | 0.33 | 0.33 |
| methyl nicotinate | 2.62 | 0.00 | 0.00 | 0.00 |
| 2-methylbutylnitrile | 1.64 | 0.00 | 0.00 | 0.00 |
| 3-methyl-,syn-butyl aldoxime | 1.31 | 0.00 | 0.00 | 0.00 |
| 3-methylbutylnitrile | 1.31 | 0.00 | 0.00 | 0.00 |
| 3-methyl-,anti-butyl aldoxime | 0.98 | 0.00 | 0.00 | 0.00 |
| nitro-2-methylbutane | 0.98 | 0.00 | 0.00 | 0.00 |
| 2-aminobenzaldehyde | 0.66 | 0.00 | 0.00 | 0.00 |
| 2-butanone oxime | 0.66 | 0.00 | 0.00 | 0.00 |
| 2-methylbutyraldoxime | 0.66 | 0.00 | 0.00 | 0.00 |
| 2-phenylnitroethane | 0.66 | 0.00 | 0.00 | 0.00 |
| 3-methylbutyronitrile | 0.66 | 0.00 | 0.00 | 0.00 |
| benzyl isonitrile | 0.66 | 0.00 | 0.00 | 0.00 |
| isobutyraldoxime | 0.66 | 0.00 | 0.00 | 0.00 |
| nitro-3-methylbutane | 0.66 | 0.00 | 0.00 | 0.00 |
| 3-formylpyridine | 0.33 | 0.33 | 0.00 | 0.00 |
| 2-methoxy3-isopropylpyrazine | 0.33 | 0.00 | 0.00 | 0.00 |
| 2-methylbutyronitrile | 0.33 | 0.00 | 0.00 | 0.00 |
| 3-methylbutanal oxime | 0.33 | 0.00 | 0.00 | 0.00 |
| 3-(methylthio)-propanenitrile | 0.33 | 0.00 | 0.00 | 0.00 |
| 4-methyl-5-vinylthiazole | 0.33 | 0.00 | 0.00 | 0.00 |
| benzyl cyanide | 0.33 | 0.00 | 0.00 | 0.00 |
| benzyl isocyanide | 0.33 | 0.00 | 0.00 | 0.00 |
| isovaleronitril | 0.33 | 0.00 | 0.00 | 0.00 |
| methyl (N-methyl)anthranilate | 0.33 | 0.00 | 0.00 | 0.00 |
| n-methyl aniline | 0.33 | 0.00 | 0.00 | 0.00 |
| phenyl acetaldoxime | 0.33 | 0.00 | 0.00 | 0.00 |
| **SULPHUR CONTAINING COMPOUNDS** | **3.61** | **1.31** | **0.66** | **0.66** |
| dimethyldisulphide | 2.95 | 0.33 | 0.00 | 0.00 |
| dimethyltrisulphide | 1.97 | 0.33 | 0.00 | 0.00 |
| dimethyltetrasulphide | 1.64 | 0.66 | 0.00 | 0.00 |
| 2,4-dithiapentane | 1.31 | 0.00 | 0.00 | 0.00 |
| 2,3,5-trithiahexane | 0.98 | 0.00 | 0.00 | 0.00 |
| 2,3,4,6-tetrathiaheptane | 0.66 | 0.00 | 0.00 | 0.00 |
| 2,3,5,6,8-pentathianonane | 0.66 | 0.00 | 0.00 | 0.00 |
| 2,4,5,7-tetrathiaoctane | 0.66 | 0.00 | 0.00 | 0.00 |
| 2-thiapropane-1-thiol | 0.33 | 0.00 | 0.00 | 0.00 |
| dimethyl sulphone | 0.33 | 0.00 | 0.00 | 0.00 |
| dimethyltrithiocarbonate | 0.33 | 0.00 | 0.00 | 0.00 |
| **MISCELLANEOUS** | **20.33** | **0.66** | **0.33** | **0.00** |
| napthalene | 8.20 | 0.33 | 0.33 | 0.00 |
| cis-jasmone | 6.56 | 0.00 | 0.00 | 0.00 |
| trans-jasmone | 2.62 | 0.00 | 0.00 | 0.00 |
| 2-methyl-3-buten-2-ol | 1.31 | 0.00 | 0.00 | 0.00 |
| 3-methyl-3-buten-1-ol | 0.98 | 0.00 | 0.00 | 0.00 |
| 3-methyl-3-butenyl acetate | 0.66 | 0.00 | 0.00 | 0.00 |
| conophthorine (trans-7-methyl-1,6-dioxaspiro[4,5]decane) | 0.66 | 0.00 | 0.00 | 0.00 |
| methyl 3-methyl-2-butenoate | 0.66 | 0.00 | 0.00 | 0.00 |
| methyl 3-pentenoate | 0.66 | 0.00 | 0.00 | 0.00 |
| 2-methyl-2-vinyl-5-oxo-tetrahydrolactone (furan) | 0.33 | 0.00 | 0.00 | 0.00 |
| 3-methylbutyl acetate | 0.33 | 0.00 | 0.00 | 0.00 |
| (E)-7-methyl-1,6-dioxaspiro[4.5]decane | 0.33 | 0.00 | 0.00 | 0.00 |
| jasmone | 0.33 | 0.00 | 0.00 | 0.00 |
| δ-decalactone | 0.33 | 0.00 | 0.00 | 0.00 |
